# QTL mapping and transcriptome analysis of seed germination under PEG-induced water stress in *Lactuca* spp.

**DOI:** 10.1038/s41598-024-77972-9

**Published:** 2024-11-07

**Authors:** Sadal Hwang, Ivan Simko, Beiquan Mou

**Affiliations:** United States Department of Agriculture, Agricultural Research Service, Sam Farr United States Crop Improvement and Protection Research Center, Salinas, CA USA

**Keywords:** Genetics, Plant sciences

## Abstract

The impact of limited water availability on lettuce growth has been well documented. However, the mechanisms by which lettuce controls seed germination under water stress remain unknown. Germination percentage was evaluated in the cv. Salinas (*Lactuca sativa*) (*L. sativa*) × US96UC23 (*Lactuca serriola*) (*L. serriola*) recombinant inbred line (RIL) population and USDA germplasm collection using 10% polyethylene glycol (PEG). About 50% of both populations displayed less than 90% germination. The average broad-sense heritability (*H*^2^) for germination percentage was 0.81 across both populations. Two quantitative trait loci (QTL) for germination percentage were identified on chromosomes 4 and 8 in the RIL population. The RNA-Seq and network analyses of wild lettuce, US96UC23, were performed using the control (distilled water, dH_2_O) and treatment (10% PEG) datasets. The number of differentially expressed genes (DEGs) was 4,095. The top 20 gene ontology (GO) terms and Kyoto Encyclopedia of Genes and Genomes (KEGG) pathways were assessed by enrichment analysis. The consensus network analysis captured 44 modules. Gene networks were constructed for the top 20 hub genes in 10 significant modules from each dataset. This study comprehensively explains QTL, GO terms, KEGG pathways, and gene networks associated with lettuce seed germination under osmotic stress.

**Supplementary Information:**

The online version contains supplementary material available at 10.1038/s41598-024-77972-9.

## Introduction

Lettuce (*L. sativa*) is one of the major vegetable crops worldwide. It was presumed that the wild ancestor of lettuce was *L. serriola* and originated in the Mediterranean Rim^1,2^. The U.S. is the second-largest lettuce producer after China^3^. California is a hub state to supply over 70% of U.S. lettuce^4^. According to the U.S. Drought Monitor^5^, the Palmer Drought Severity Index has indicated exceptional drought status in over 50% of California for decades, affecting both major lettuce production areas, the Central Coast and Central Valley. Lettuce production in California requires a high volume of water from germination to harvest, even with drip irrigation^6,7^. Due to the economic aspect of drip irrigation, farmers consume more water in sprinkler or furrow irrigation while water resources in the aquifers continue to decline.

Water stress diminishes crop yield by shifting metabolism, leading to accelerated senescence at the whole plant. Previous studies elucidated that *L. serriola* was resistant to water stress at early (four weeks after germination) and mature stages^8–10^. The *L. serriola* has unique morphological characteristics that maintain high water use efficiency under water deficit conditions. The lateral roots and taproot of *L. serriola* grow longer to access deep soil moisture, whereas cultivated lettuce has shallow lateral roots and taproots^11^. The leaves of *L. serriola* have many trichomes^12^. After bolting, the cauline leaves of *L. serriola* adjust to the vertical direction and reduce leaf temperature and transpiration^13,14^.

Germination is a complex process requiring enough water in optimized environmental conditions. Seed germination is sensitive to limited water acquisition^15,16^. A severe water shortage during germination considerably affects the quality of seedlings and yield^17^. The increment of osmotic stress reduces the final germination percentage and the water content in the seeds^18^. Water stress constrains water absorption by seeds and defers protein synthesis by affecting the transfer of nutrient reserves^19,20^. The optimal temperature range for *L. serriola* germination is 15 °C to 25 °C, with a broader range of 10 °C to 35 °C^21^. The life cycle and wide temperature range of *L. serriola* indicate that it has little primary dormancy^22^.

The seeds reduce the water potential (*Ψ*), turgor pressure, and cell elongation under water stress. PEG has been widely employed to simulate water-deficit conditions. Its heavy molecular weight and non-toxic nature maintain a consistent osmotic potential^23–25^. Previous PEG studies demonstrated that the germination percentage of *L. serriola* reduced severely as *Ψ* decreased up to − 0.5 MPa^26^. The cultivated lettuce germinated at *Ψ* as low as − 0.41 MPa^27^.

Many genetic mapping studies identified QTL for water stress-related traits in the cv. Salinas (*L. sativa*)^28^ × US96UC23 (*L. serriola*) interspecific RIL population^29–31^. Most QTL studies on lettuce seed germination have been related to thermo-dormancy at high temperature^32,33^. The locus, *Htg6.1*, was mapped and associated with the biosynthesis of abscisic acid (ABA). However, it remains unknown how seed germination under water stress is regulated in lettuce. Hence, this study aimed to (1) evaluate the germination percentage in the cv. Salinas × US96UC23 RIL population and USDA germplasm collection under osmotic stress with 10% PEG, (2) identify QTL for germination percentage in the RIL population, and (3) investigate the transcript and gene network for seed germination in US96UC23 under control (dH_2_O) and treatment (10% PEG) conditions as a case study.

## Results

### Trait evaluation

**Fig. 1:**
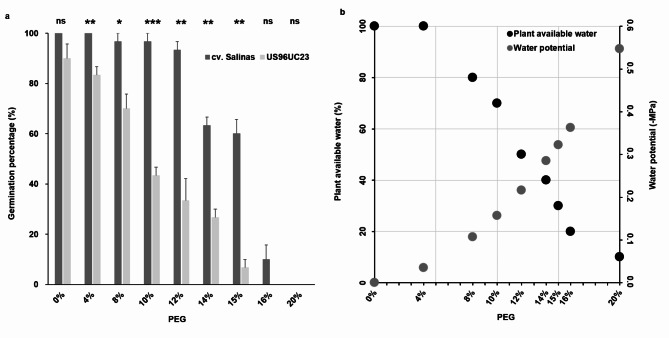
(**a**) Comparison of germination percentages between cv. Salinas and US96UC23. The symbols on the top of the bar plot, ns, *,**, and ***, indicated *P* > 0.05 (not significant), *P* < 0.05, *P* ≤ 0.01, and *P* ≤ 0.001, respectively. (**b**) Relationship between PAW and *Ψ* at different PEG concentrations. The *Ψ* was determined using the equation^23^. The PAW in sandy loam soil was estimated using the *Ψ* equation^23^ and field capacity from previous hydrology studies^55,56^.

The germination percentages between two genotypes, cv. Salinas and US96UC23, were compared at nine PEG concentrations (Fig. 1a). Two-way analysis of variance (ANOVA) indicated that the effects of genotype and PEG were significant (*P*-values ≤ 1.69e−07). The *t*-tests showed significant differences between genotypes from 4 to 15% PEG. The mean differences between genotypes were 36.6–60% at 10–15% PEG, implying a high likelihood of identifying QTL in the cv. Salinas × US96UC23 RIL population. The germination percentage between genotypes was not different at 0% PEG (dH_2_O). The cotyledons from the seeds of both cv. Salinas and US96UC23 emerged with extended hypocotyl and radical at dH_2_O (Supplementary Fig. 1). US96UC23 was more sensitive to water stress than cv. Salinas. The germination percentage of US96UC23 was less than 50% at 10% PEG. No germination was found at 20% PEG. The *Ψ* ranged from 0 to − 0.55 MPa at nine PEG concentrations (Fig. 1b). The *Ψ* and plant available water (PAW) were − 0.16 MPa and 70% at 10% PEG, respectively. The PAW was about 50% at 12% PEG (*Ψ* = − 0.22 MPa) and 20% at 16% PEG (*Ψ* = − 0.36 MPa). As mild water stress treatment, 10% PEG was used for the subsequent germination experiments.

The germination percentage of the RIL population and USDA germplasm collection (Supplementary Data 1) was evaluated at 10% PEG. The frequency distributions of germination percentage were analyzed for both populations (Supplementary Fig. 2). The histograms of both populations had left-skewed distributions with heavy tails. The median germination percentages in the RIL population and USDA germplasm collection were 80% and 90%, respectively. The germination percentage was less than 90% in 66% of the RILs and 44% of the USDA accessions, indicating that seed germination in lettuce was sensitive to water stress.

The germination percentage of the RIL population and USDA germplasm collection did not follow normal distributions (*P*-values < 2.2e−16). Therefore, the Kruskal-Wallis (*H*) test was conducted in both populations (Table 1). The genotypes (RIL and accession) had a highly significant effect in both populations (*P*-values ≤ 9.92e−13). The broad-sense heritabilities for germination percentage were 0.825 and 0.801 in the RIL population and USDA germplasm collection, respectively. The narrow-sense heritability (*h*^2^) for germination percentage was 0.63.

**Table 1:** Kruscal-Wallis test of germination percentage at 10% PEG in the cv. Salinas × US96UC23 RIL population and USDA germplasm collection. ^a^Degree of freedom.

Source	*df* ^a^	Sum of squares	Mean squares	*H* test	*P*-value
cv. Salinas × US96UC23 RIL population					
RIL	212	2892687.25	13644.75	390.65	9.92e−13
Error	213	508,717	2388.34		
Total	425	3401404.25			
USDA germplasm collection					
Accession	523	40587941.88	77606.01	952.11	< 2.2e−16
Error	524	8090195.25	15439.30		
Total	1047	48678137.13			

### Genetic map analysis

The public genetic map of the cv. Salinas × US96UC23 RIL population was originally developed with 13,943 single-position polymorphism markers (SSPs) and reconstructed by discarding erroneous and redundant SSPs. The segregation ratio tests indicated that 553 SPPs on chromosomes 3 (533), 4 (1), 5 (3), 7 (3), and 8 (13) did not fit a 1:1 ratio of cv. Salinas: US96UC23 alleles (Supplementary Fig. 3). On chromosomes 3, 7, and 8, 549 SPPs showed a higher ratio of cv. Salinas allele. All segregation-distorted SPPs were discarded. Duplicate and monomorphic 8,608 SPPs were also removed. The relationship between recombination frequency (*rf*) and the logarithm of the odds (LOD) was evaluated in all SPP pairs (Supplementary Fig. 3). All SPP pairs were tested to find a SPP with switched alleles, and no such SPP was detected.

The final genetic map was reconstructed with 5,322 SPPs (Supplementary Fig. 4). The total length of the map was 1,969.8 cM and 1.2 times longer than the previous public one. The average distance between adjacent markers was 0.4 cM. The maximum distance between two SPPs, BULA_0 and AYXI_0, was 53.7 cM due to the deletion of segregation-distorted markers at the long arm of chromosome 3.

### QTL analysis

QTL for germination percentage at 10% PEG were identified in the cv. Salinas × US96UC23 RIL population. Two QTL, ANIM_0 and AXDH_0, were identified on chromosomes 4 and 8 using different QTL models, respectively (Supplementary Fig. 4 and Table 2). ANIM_0 explained 7.3% to 8.8% of the total variance of the germination percentage. The R^2^ values of AXDH_0 were 8.3% and 5.7% in composite interval mapping (CIM) and multiple interval mapping (MIM), respectively. The QTL effects of both QTL ranged from 6.1% to 7.86%. Cv. Salinas and US96UC23 provided favorable alleles for a higher germination percentage at ANIM_0 and AXDH_0, respectively. The significant interaction between both QTL was detected in the MIM model (LOD = 2.91) with a less stringent Bayesian information criterion (BIC). The interaction effect was 6.08%, and the R^2^ value of the interaction was 5.3%. Considering A and B alleles originated from cv. Salinas and US96UC23, respectively, the germination percentages of four genotype groups, AA/AA, AA/BB, BB/AA, and BB/BB, were compared. (Supplementary Fig. 5). Each genotype group consisted of the first two alleles from ANIM_0 and the last two from AXDH_0. The *H* test showed that the germination percentages of the four groups were significantly different (*P* = 2.29e−06). The Dunn tests indicated that the germination percentage of BB/AA was about 1.5 times lower than the other three genotype groups.

**Table 2:** QTL for germination percentage at 10% PEG in the cv. Salinas × US96UC23 RIL population.

Model^a^	QTL marker	Chromosome	Position^b^	LR^c^	LOD	R^2^	Effect^d^	Favorable allele^e^	Criterion^f^
SMA	ANIM_0	4	197.6	16.22	3.53	8.6	7.14	cv. Salinas	1.03e−04
IM	ANIM_0	4	197.6	16.13	3.51	7.3	7.10	cv. Salinas	1.13e−04
CIM	ANIM_0	4	197.6	22.47	4.88	8.8	7.86	cv. Salinas	1.55e−04
	AXDH_0	8	129.4	21.30	4.63	8.3	7.66	US96UC23	1.55e−04
MIM	ANIM_0	4	197.6	16.11	3.50	7.3	7.12	cv. Salinas	BIC-M3
	ANIM_0	4	197.7	19.23	4.18	7.5	7.34	cv. Salinas	BIC-M2
	AXDH_0	8	129.5	13.53	2.94	5.7	6.10	US96UC23	BIC-M2

^a^SMA (single marker analysis), IM (interval mapping), and CIM (composite interval mapping) were used as single QTL models. The MIM (multiple interval mapping) was used as a multiple QTL model.

^b^Genetic map position (cM) in the cv. Salinas × US96UC23 RIL population.

^c^Likelihood ratio (LR) was equal to 4.6 x LOD.

^d^QTL effect was defined as one-half the mean of US96UC23 alleles minus one-half the means of cv. Salinas alleles as an average effect of allele substitution or breeding value/2.

^e^Favorable allele was defined as the allele giving a higher germination percentage.

^f^FDR-adjusted *P*-values and BIC were used for single and multiple QTL models.

Candidate genes were screened within the 95% confidence intervals (CIs) of both QTL (Supplementary Table 1). ANIM_0 was physically located between 234,370,421 bp and 234,371,251 bp on chromosome 4. No annotated lettuce gene was linked to ANIM_0. However, the homologous *Arabidopsis thaliana* (*A. thaliana*) gene, AT5G21280.1, was found in the region where ANIM_0 was positioned. AXDH_0 was located between 124,467,301 bp and 124,469,258 bp on chromosome 8. The lettuce gene, Lsat_1_v5_gn_8_85600.1 (LS21897), was linked to AXDH_0 and homologous to AT3G59530.3.

### Trait evaluation of US96UC23

For RNA-Seq and network analyses, the germination percentage of US96UC23 was assessed under control (dH_2_O) and treatment (10% PEG) conditions. The *t*-test indicated that the mean difference (35.3%) in germination percentage between the control and treatment groups was highly significant (*P* = 1.85e−06). The average germination percentages of US96UC23 were 88% in the control group and 52.7% in the treatment group.

### DEG and enrichment analyses

After filtering available fragments per kilobase of transcript per million mapped reads (FPKM), GO, and KEGG data in US96UC23, 27,245 genes were used for DEG and enrichment analyses (Supplementary Data 2). The gene IDs for 27,245 genes were created by combining the letters “LS” and a 5-digit number. These IDs were used to depict lettuce genes instead of long gene model names in the lettuce reference genome.

DEGs were identified to ascertain significantly upregulated and downregulated genes using the FPKM data from the control and treatment datasets. The FPKM data was evaluated as the normalized transcript levels (Supplementary Data 2 and Supplementary Fig. 6). Given all the replicates of US96UC23 across both datasets, 23,742 FPKM values were averagely greater than 0. The overall mean of FPKM values was 32.68, and FPKM values ranged from 0 to 30,747. The standard deviation of FPKM values ranged from 150.51 to 434.03. The *t*-test showed a significant FPKM mean difference (3.71) between the control and treatment datasets (*P* = 5e−04). The average FPKM values for the control and treatment datasets were 34.59 and 30.9, respectively. The total number of DEGs was 4,095, of which 2,750 were upregulated and 1,345 were downregulated (Supplementary Data 2 and Supplementary Fig. 7).

The GO term and KEGG pathway, which were annotated in the highest number of DEGs, were identified. A total of 2,839 GO terms and 126 KEGG pathways were annotated in 27,245 genes. Three GO terms, biological process (GO:0008150), nucleus (GO:0005634), and molecular function (GO:0003674), were annotated in the largest number of DEGs in each GO class, biological process, cellular component, and molecular function, respectively (Supplementary Fig. 8). The KEGG pathway, plant hormone signal transduction (ko04075), was annotated in the largest number of DEGs (Supplementary Fig. 9).

**Fig. 2:**
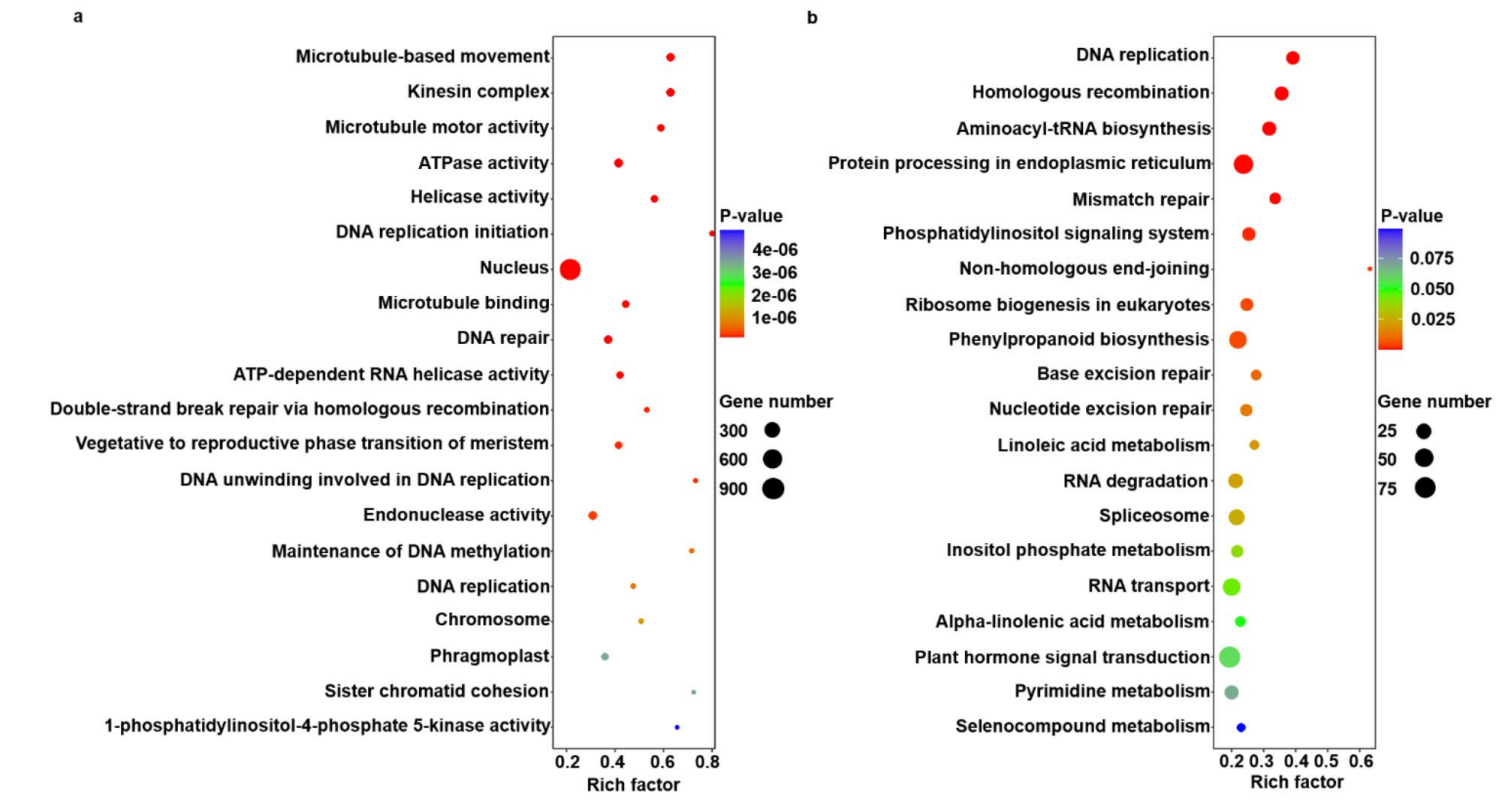
(**a**) Top 20 GO terms in GO enrichment analysis. (**b**) Top 20 KEGG pathways in KEGG enrichment analysis.

Enrichment analysis was performed to investigate the top 20 GO terms and KEGG pathways and collect biological data, such as GO IDs and reference pathways. The top 20 GO terms were identified by GO enrichment analysis (Fig. 2a). The *P*-values of the top 20 GO terms were less than 4.79e−06. Microtubule-based movement (GO:0007018) was the most significant (*P* = 3.19e−19). Nucleus (GO:0005634) had the lowest rich factor (0.17), whereas DNA replication initiation (GO:0006270) indicated the highest rich factor (0.78). The top 20 KEGG pathways were identified by KEGG enrichment analysis (Fig. 2b). However, the *P*-values of four KEGG pathways, alpha-linolenic acid metabolism (ko00592), plant hormone signal transduction (ko04075), pyrimidine metabolism (ko00240), and selenocompound metabolism (ko00450), were slightly greater than 0.05. DNA replication (ko03030) was the most significant (*P* = 2.08e−08). Plant hormone signal transduction (ko04075) had the lowest rich factor (0.17), and non-homologous end-joining (ko03450) showed the highest rich factor (0.63). A total of 1,804 DEGs were associated with the top 20 GO terms and KEGG pathways. Additionally, 228 DEGs overlapped between the top 20 GO terms and KEGG pathways (Supplementary Data 3).

### Network construction

**Fig. 3:**
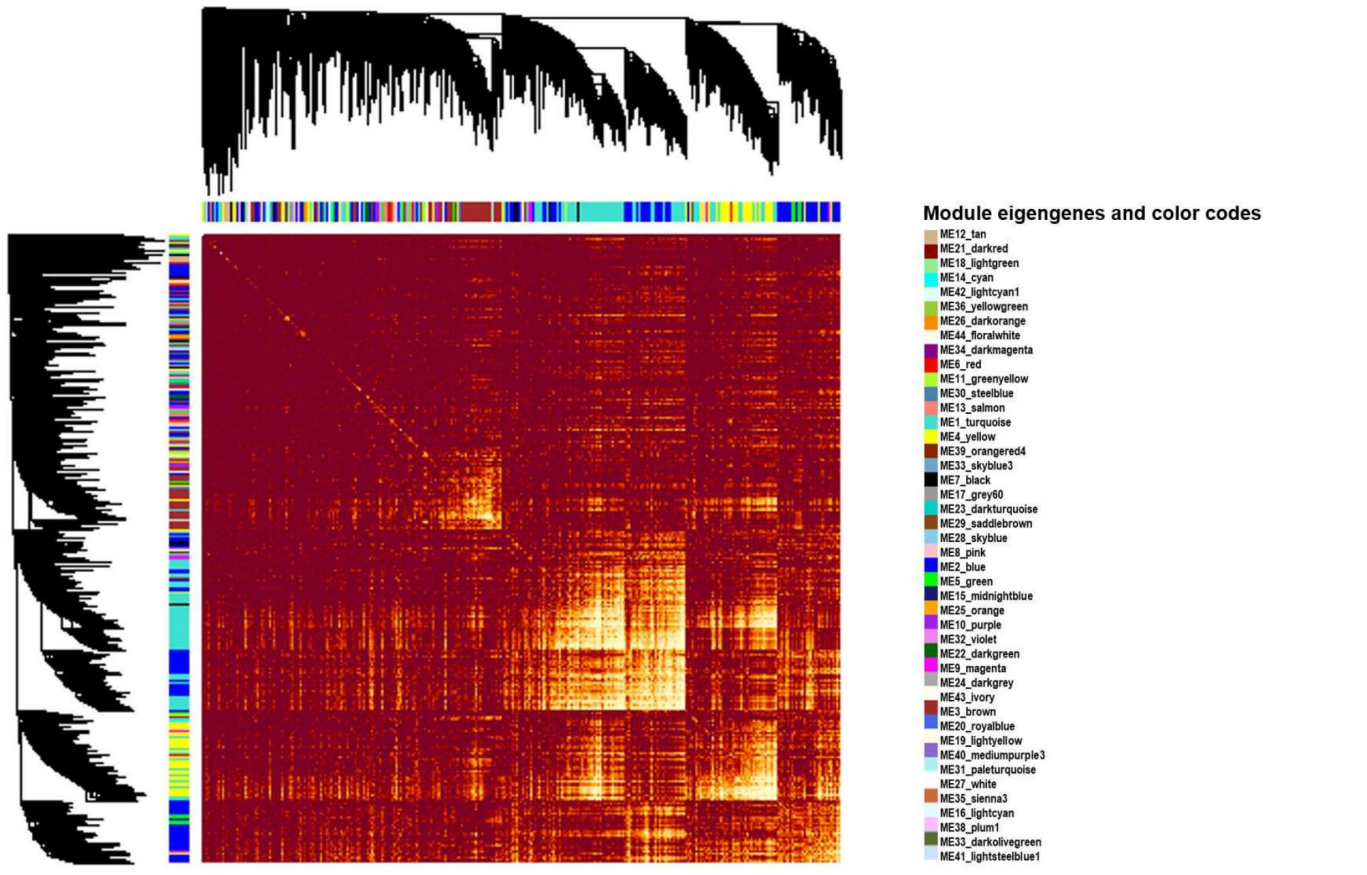
Topological overlap matrix among all genes in the gene network. Darker dark red represented low overlap, while light yellow represented high overlap. The assigned modules with colors and dendrograms are shown along the left side and top. In the clustered dendrogram (tree), branch groups were densely interconnected with highly co-expressed genes. The vertical line of each branch corresponded to a gene.

The network was constructed using the FPKM data from the control and treatment datasets in US96UC23 (Supplementary Data 4). A total of 26,071 genes, including 99.4% of DEGs, were used for network construction, excluding genes with either 0 or many missing FPKM values. The selected soft threshold power (*β*) was 6, considering the scale-free topology model and connectivity range (Supplementary Fig. 10). The R^2^ values of topology models were 0.8 and 0.9 in the control and treatment datasets at *β* = 6, respectively. The consensus network analysis identified 44 modules across both datasets, implying that 44 module eigengenes (MEs) were obtained from each dataset (Supplementary Data 4 and Fig. 3). The number of genes in each module ranged from 6,651 (module 1) to 47 (module 44). The 48 genes in the grey-colored module were not assigned to any of the 44 identified modules in the consensus network analysis. The module in grey was nominally designated as module 0, and the ME of module 0 was designated as ME0.

### Eigengene significance, module significance, and module membership

Eigengene significance was evaluated to detect significant modules by testing the correlation coefficient between the germination percentage and ME in a module (Supplementary Table 2). Only ME0 was significantly correlated with the germination percentage in the control dataset (*P* = 1.4e−2). The eigengene significance between ME0 and the germination percentage was − 0.63. The MEs of ten modules 1, 4, 6, 9, 17, 19, 24, 36, 37, and 38 were significantly correlated with the germination percentage in the treatment dataset (*P*-values < 0.05), indicating that the ten modules were significant for the network study. The absolute eigengene significance in 10 MEs was greater than 0.53. Three MEs, ME9, ME36, and ME38, indicated positive eigengene significance. ME38 showed the highest correlation with the germination percentage (*P* = 2e−4).

Module significance was estimated as the average absolute gene significance (GS) for all genes in a module to support the relationship between eigengene significance and module significance (Supplementary Table 2). Module 0 in the control dataset showed the highest module significance (0.49). Ten modules with significant MEs exhibited higher module significance than others in the treatment dataset. The module significance of the ten modules ranged from 0.33 to 0.53. The highest module significance was 0.53 in module 38.

Module membership (MM) was evaluated to determine gene membership by testing the correlation coefficient between a gene and ME in a module (Supplementary Table 2). The MM of each gene in a module, except for module 0, was significant (*P*-value < 0.05). The average absolute MM across all modules was 0.72 and 0.67 in the control and treatment datasets, alluding that the genes in a module had significant MM and high connectivity.

### Gene significance

The GS of a gene was evaluated to determine if the gene was significantly correlated with the germination percentage in the control and treatment datasets (Supplementary Data 4 and Supplementary Fig. 11). The total number of significant genes was 6,378, of which 1,748 DEGs (27.4%) were included. A total of 319 and 5,451 genes were significant in the control and treatment datasets, respectively. The GS of LS00896 in module 2 was − 0.77, indicating the most negative relationship (*P* = 1.36e−3) with the germination percentage in the control dataset. The GS of LS09012 in module 1 was − 0.914, with the highest correlation (*P* = 1.93e−6) in the treatment dataset. As a DEG, LS20107 in module 4 had the lowest meta *P*-value (6.76e−6) across both datasets. The Venn diagram showed that 30 significant genes commonly overlapped in each dataset and across both datasets (Supplementary Data 5 and Supplementary Fig. 12). Eight DEGs, LS04614, LS06392, LS11691, LS23172, LS01020, LS20107, LS20239, and LS21606, were observed from 30 significant genes in modules 1 (3), 2 (1), 3 (1), 4 (1), 17 (1), and 24 (1), respectively.

### Gene network

**Fig. 4:**
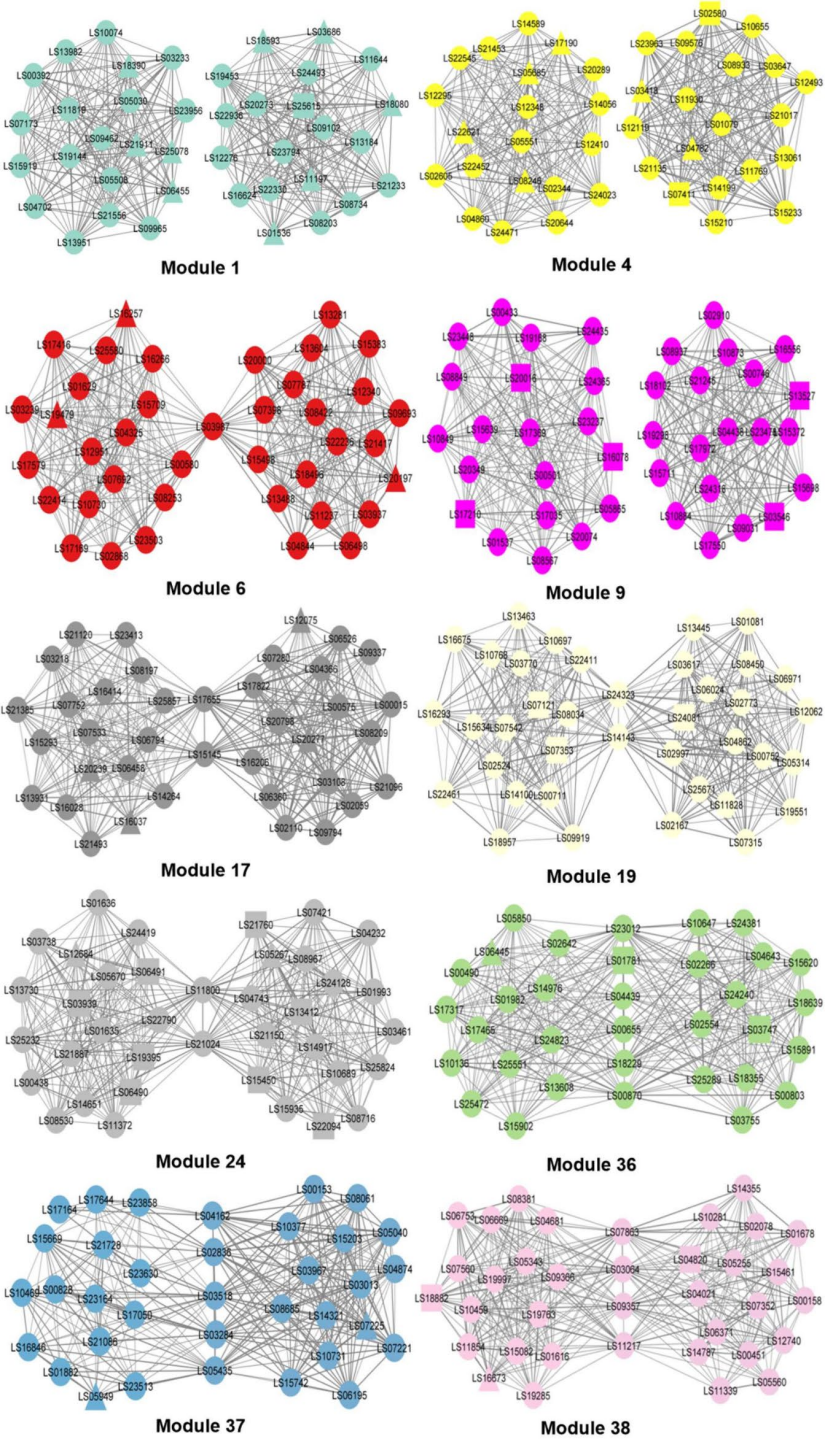
Gene networks for the top 20 hub genes in 10 modules from the control and treatment datasets. The organic layout style was applied for the construction of gene networks. The gene networks of control and treatment datasets were on the left and right in each module. The node colors of gene networks corresponded to the assigned module colors. The round-shaped nodes were not DEGs. The triangle- and rectangle-shaped nodes indicated upregulated and downregulated DEGs in each gene network, respectively. The node size by node degree was not considered, highlighting node color and gene ID in each gene network. However, a degree of edge was given in each gene network. The nodes in the middle of two gene networks in each module were commonly linked between the gene networks of both datasets.

Eigengene significance tests found ten significant modules in the treatment dataset (Supplementary Table 2). Although no significant module was in the control dataset, the gene networks in the ten modules of the control dataset were also considered. Therefore, gene networks were constructed for the top 20 hub genes in each of the ten modules to compare the gene networks of both the control and treatment datasets (Fig. 4 and Supplementary Data 6 and 7).

Twenty-two genes, LS03987, LS15145, LS17655, LS14143, LS24323, LS11800, LS21024, LS00870, LS01781, LS23012, LS00655, LS04439, LS18229, LS02836, LS03284, LS03518, LS05435, LS04162, LS03064, LS11217, LS07863, and LS09357, were commonly connected between the gene networks of both datasets in modules 6 (1), 17 (2), 19 (2), 24 (2), 36 (6), 37 (5), and 38 (4), respectively. Four genes, LS15145, LS21024, LS23012, and LS02836, were found to have the highest node degree and betweenness centrality in modules 17, 24, 36, and 37, respectively. These four genes had the most connections with other genes and played a critical role in exerting the interaction of other genes in the gene network.

A small number of DEGs were found to be involved in all gene networks. One DEG, LS01781, was connected between the gene networks of both datasets in module 36. In the control dataset, ten DEGs, LS21911, LS19479, LS20016, LS20239, LS07353, LS06490, LS19395, LS01781, LS16673, and LS18882, indicated the highest node degree or betweenness centrality in modules 1 (1), 6 (1), 9 (1), 17 (1), 19 (1), 24 (2), 36 (1), and 38 (2), respectively. Four DEGs, LS17190, LS07121, LS03939, and LS05949, showed the highest neighborhood connectivity in modules 4, 19, 24, and 37, respectively. These four DEGs represented the top average connectivity of all neighbors as a local centrality measure in the gene network. In the treatment dataset, 9 DEGs, LS03686, LS18593, LS03546, LS13527, LS20277, LS24081, LS05267, LS21760, and LS07225, displayed the highest node degree and betweenness centrality in modules 1 (2), 9 (2), 17 (1), 19 (1), 24 (2), and 37 (1), respectively. Thirteen DEGs, LS11197, LS25615, LS02580, LS03418, LS04782, LS07411, LS20197, LS03546, LS13527, LS12075, LS01781, LS03747, and LS07225, had the highest neighborhood connectivity in modules 1 (2), 4 (4), 6 (1), 9 (2), 17 (1), 36 (2) and 37 (1), respectively.

Based on the enrichment analysis (Fig. 2), some genes in the gene networks were annotated with the top 20 GO terms and KEGG pathways. In the control dataset, four genes, LS07173, LS05685, LS05949, and LS11854, were in modules 1, 4, 37, and 38, respectively. In the treatment dataset, seven genes, LS21135, LS13281, LS10884, LS24318, LS20277, LS07421, and LS00803, were in modules 4 (1), 6 (1), 9 (2), 17 (1), 24 (1), and 36 (1), respectively. These genes exhibited the highest node degree, betweenness centrality, or neighborhood connectivity in the gene network.

## Discussion

The segregation-distorted 553 SPPs were detected by the stringent criterion in the cv. Salinas × US96UC23 RIL population (Supplementary Fig. 3). The segregation distortion might result from preferential gametic transmission in interspecific populations^34,35^. The integrated map study demonstrated that markers in similar chromosome regions indicated segregation distortion in different F_2_ interspecific populations rather than intraspecific ones^36^. However, the direction of marker distortion in these different F_2_ interspecific populations did not align with the cv. Salinas × US96UC23 RIL population. Our results have been supported by another previous study conducted on the same RIL population^37^. Interestingly, on chromosome 3, 533 segregation-distorted SPPs were found between two flanking markers in the cv. Salinas × US96UC23 RIL population, showing a higher ratio of cv. Salinas alleles in a 122 Mb physical map interval (Supplementary Fig. 4). The segregation-distorted SPPs could affect the identification of covariates in the CIM and MIM models for QTL analysis. Therefore, all distorted markers were discarded for QTL analysis in this study.

Candidate genes in lettuce and their homologous *A. thaliana* genes were identified by narrowing down from three data sources: (1) the CIs of QTL (Supplementary Table 1), (2) duplicate DEGs between the top 20 GO terms and KEGG pathways by enrichment analysis (Supplementary Data 3), and (3) network analysis (Supplementary Data 5, 6, and 7). Based on previous studies, we highlighted some notable *A. thaliana* genes associated with seed germination in each data source mentioned above.

In the CIs of QTL (Supplementary Table 1), three *A. thaliana* genes, AT3G20290.3, AT5G43020.1, and AT5G56550.1, were homologous to Lsat_1_v5_gn_4_127040.1, LS10622, and LS21902, respectively. These *A. thaliana* genes were related to stress or hormone. Endocytosis and exocytosis were complementary membrane trafficking processes to maintain plasma membrane integrity and stress tolerance^38^. The immunolocalization analyses with pectin and actin revealed that AT3G20290.3 was endocytosed in the cell walls of the seed embryo^39^ and root hairs^40^ once the seed imbibition started. The T-DNA insertion lines revealed that AT5G43020.1 was involved in root development defects and signal transduction pathways related to hormone and abiotic stress^41^. AT5G56550.1 suppressed ABA signaling by regulating histone deposition at the *ABI4* promoter under environmental stress^42^. As a transcription factor (TF), *ABI4* played a pivotal role in the ABA pathway by repressing cytokinin-inducible genes and promoting gibberellin degradation at the post-germination stage^43^.

In the list of duplicate DEGs between the top 20 GO terms and KEGG pathways by enrichment analysis (Supplementary Data 3), five *A. thaliana* genes, AT5G03730.1, AT5G58760.1, AT1G48130.1, AT2G26040.1, and AT2G38310.1, were homologous to LS01272, LS14778, LS16627, LS12480, and LS20279, respectively. These *A. thaliana* genes were related to abiotic stress or cross-talk in hormones. The C_2_H_4_-susceptible mutant of AT5G03730.1 reduced sensitivity to ABA, which was promoted by cytokinin in the signaling of seed germination^44^. AT5G58760.1 encoded the damaged DNA binding protein 1, a component of the DE-ETIOLATED 1 (DET1) complex. The *det1* mutant resisted osmotic and salt stress in germination^45^. AT1G48130.1 played essential roles in the biosynthesis of lignin, flavonoid, and coumarin in the phenylpropanoid pathway. The mutant of AT1G48130.1 suppressed seed dormancy by reducing ABA and increasing gibberellic acid (GA) during germination^46^. Both AT2G26040.1 and AT2G38310.1 transcribed the ABA-binding receptors. The overexpression of ABA receptor kinases inhibited seed germination and enhanced drought resistance^47^.

Five *A. thaliana* genes, AT5G48670.1, AT2G14210.2, AT5G11460.1, AT5G66880.1, and AT1G12920.1, were related to TF or hormone signal transduction. AT5G48670.1 was homologous to LS06419 in the GS of network analysis (Supplementary Data 5). In the gene networks for the top 20 hub genes (Supplementary Data 6 and 7), four other *A. thaliana* genes were homologous to LS19285, LS06195, LS07421, and LS02836, respectively. AT5G48670.1 was a MADS-box TF in the central gene regulatory network that controlled the expression of downstream genes in cell development and function^48^. AT2G14210.2 was a member of MADS-box TFs. The overexpressing lines were hypersensitive to ABA, salt, and osmotic stress in the seed germination^49^. AT5G11460.1 interacted with sucrose nonfermenting-1-related protein kinase 1 (SnRK1), a central plant growth regulator. During energy starvation, the gene was induced and repressed SnRK1 by increasing the level of SnRK1α1 as the major subunit of SnRK1. The mutant of AT5G11460.1 accumulated more SnRK1α1 and inhibited germination under favorable growth conditions^50^. AT5G66880.1 encoded SnRK2.3. The gene was induced by salt or osmotic stress and was involved in ABA signaling^51^. The mutation of substrates, which interacted with SnRK2.3, increased ABA signaling. AT1G12920.1 was plainly induced by glucose and hypersensitive to glucose in germination. The overexpressing lines enhanced sensitivity to the inhibitor of GA signal pathways^52^.

It is important to carefully select the stage and target tissue when collecting samples for RNA-Seq analysis. Like the seeds in Supplementary Fig. 1, we observed that most seeds from the RIL population and USDA germplasm collection germinated well after 4 days under control conditions. Hence, we decided to take samples 4 days after seed imbibition. Even mild water stress at 10% PEG resulted in wide variation in the germination of US96UC23 seeds, including well-germinated seeds, as shown in Supplementary Fig. 1. The relationships among MEs exhibited that the treatment dataset showed a wider range of correlation coefficients than the control dataset (Supplementary Fig. 13). Collecting all seed tissues could be a disadvantage for precise RNA-Seq analysis rather than analyzing specific tissues (e.g., root, cotyledon). This study focused on a general transcriptome analysis to understand how osmotic stress affected seed germination.

Since both QTL were identified in the controlled environmental conditions, it would be essential to conduct the field test if both QTL were stable on a multi-year or -location basis. It will be critical to confirm both QTL and identify new QTL in diverse mapping populations to apply marker-assisted or genomic selection and expand gene network information. The transcriptome analysis of US96UC23 facilitated the discovery of significant GO terms, KEGG pathways, and gene networks associated with seed germination under osmotic stress in lettuce. In future studies, identifying expression QTL (eQTL) and gene networks at the population level will help build an explicit regulatory network of seed germination in lettuce.

## Methods

### Germination experiment

The PEG 8000 (Sigma-Aldrich, cat. P5413) was used to make a PEG solution using autoclaved and filtered dH_2_O. The filter paper with 85 mm diameter (Sigma-Aldrich, cat. WHA1001085) was drenched in 6 ml dH_2_O or PEG solution. Seeds were placed on a single layer of saturated filter paper in sterilized and transparent polystyrene Petri dishes with 100 mm × 20 mm size (Sigma-Aldrich, cat. P5606). The Petri dish lid had three ventilation ribs on the underside, allowing a continuous oxygen supply. The seeds were imbibed for 4 days in the GR-41L seed germination chamber (Geneva Scientific LLC, WI, USA). The white fluorescent tube lights were turned on for 9 h during the daytime, and the irradiance intensity was medium (60 micromoles/m^2^/sec)^53^. The optimal temperatures were set at 18 °C and 15 °C for the day and night^21^. Radicle emergence (RE)^54^ was measured after 4 days. The seeds with RE greater than 5 mm were counted as germinated ones. The counted seed numbers were converted to the germination percentage (%).

### Estimation of water potential and plant available water

To discern the degree of water stress by different PEG concentrations, *Ψ* and PAW were calculated. The *Ψ* was determined using the equation: *Ψ* = 1.29*C*^2^*T* − 140*C*^2^ − 4*C* (*C*: PEG concentration (g/ml) and *T*: temperature (°C))^23^. The day temperature, 18 °C, was applied for the variable *T* in the equation. The PAW in sandy loam soil was estimated using the *Ψ* equation^23^ and field capacity from previous hydrology studies^55,56^.

### Experimental design and statistical analysis

Three germination experiments were performed with a completely randomized design (CRD) from 2022 to 2023. First, cv. Salinas and US96UC23 were compared at 0% to 20% PEG in 3 replicates. Second, the F_7:8_ cv. Salinas x US96UC23 RIL population and USDA germplasm collection, consisting of 213 RILs and 524 USDA accessions (Supplementary Data 1), were evaluated at 10% PEG in 2 replicates. As parents of the RIL population, cv. Salinas and US96UC23 were also included among 524 USDA accessions. Third, for RNA-Seq and network analyses, US96UC23 was assessed in the control (dH_2_O) and treatment (10% PEG) groups, with 15 replicates in each group. Thirty seeds were used per replicate in all germination experiments. The seeds of the RILs, their parents, and the USDA accessions were harvested in 2021.

All statistical analyses were performed with R (V. 4.2.1). The residuals of the CRD linear model in each germination experiment were tested for normal distribution using the shapiro.test(). If the estimated parameter *λ* in the Box-Cox procedure^57^ did not result in a satisfactory transformation, the *H* test^58^ was conducted instead of general ANOVA. The germination percentage was converted to ranked data as a categorical variable in the *H* test.

The rank() was used to rank a value in the germination percentage data. The Kruskal.test() was used for the *H* test. The Dunn test^59^ was used as a *post hoc* test if the null hypothesis of the *H* test was rejected at α = 0.05. The dunnTest() with Bonferroni-adjusted criterion was used for the multiple comparison test. The aov() was used for general ANOVA, and the t.test() was used for two sample or paired *t*-test. The as.factor() was used to convert an independent variable into a factor. The cor.test() was used to estimate the Pearson correlation coefficient (*r*) between two variables.

### Heritability

The *H*^2^ for germination percentage at 10% PEG was estimated using the mean squares of *H* test results in the RIL population and USDA germplasm collection. The *H*^2^ was estimated as follows: *H*^2^ = *δ*_g_^2^/(*δ*_g_^2^ + (*δ*_e_^2^/*r*)) (*δ*_g_^2^: genetic variance, *δ*_e_^2^: error variance, and *r*: number of replicates). Based on the Breeder’s equation^60^, the *h*^2^ for germination percentage at 10% PEG was estimated using the RIL population and USDA germplasm collection. The USDA germplasm collection was considered the base population. When cv. Salinas and US96UC23 were selected from the base population, and their RIL population was generated, *h*^2^ was estimated as follows: *h*^2^ = *R* (response to selection)/*S* (selection differential) = (*µ*_sxu_ – *µ*_g_)/(*µ*_a_ – *µ*_g_) (*µ*_sxu_: the average germination percentage of 213 RILs in the cv. Salinas x US96UC23 population, *µ*_g_: the average germination percentage of 524 USDA accessions in the USDA germplasm collection, and *µ*_a_: the average germination percentage of cv. Salinas and US96UC23).

### Genetic map construction

The public genetic map was developed in the 213 F_7_ cv. Salinas x US96UC23 RIL population using 13,943 UniGene-based SPPs^37^. The alleles of each SPP were designated with A or B in the Affymetrix array. The A and B alleles originated from cv. Salinas and US96UC23, respectively. The R/qtl package in R^61^ was used to identify erroneous and redundant SPPs and reconstruct the final genetic map. The geno.table() was used to test the segregation ratio of each SPP using the Chi-squared test. The segregation-distorted markers were removed based on a Bonferroni-adjusted criterion. The redundant markers were deleted using the findDupMarkers() and drop.markers(). The checkAlleles() was used to identify the marker loci with erroneously switched alleles with LOD 3.0. The map distance was tested by the maximum likelihood with the Expectation-Maximization algorithm^62^. The est.map() calculated the map distance between markers based on the estimated *rf*. The Kosambi function^63^ was used for all linkage analyses. The jittermap() was used to slightly separate coincident marker positions.

### QTL analysis

WinQTLCartographer (V. 2.5.011)^64^ was used to identify QTL for germination percentage in 213 F_7:8_ cv. Salinas × US96UC23 RIL population. The average germination percentage of each RIL was estimated using the least squares (LS) means in the CRD linear model. The LS means were used for the germination percentage data in the QTL models. As a single-QTL model, single marker analysis (SMA), interval mapping (IM), and CIM were conducted. Model 6 was chosen for CIM^65^, and the number of control background markers was 5. The Forward Regression method (α = 0.05) was applied to mitigate errors from a stepwise approach and protect against model overfitting. The single-QTL models used the false discovery rate (FDR)-adjusted *P*-value (*Q*-value)^66^ to test the null hypothesis (H_0_: there is no QTL). MIM^67^ was performed as a multiple-QTL model. The MIM Forward Search method identified the initial QTL. The Refine Model performed the statistical tests of identified main QTLs and their interactions. The final model was decided based on the two BIC criteria, BIC-M2 (2ln(n)) and BIC-M3 (3ln(n)) (n: population size). The Window size and Walk speed were 1 cM for all QTL models.

### RNA-Seq analysis

All germinated seeds (RE > 5 mm) of US96UC23 were collected from each replicate in the control and treatment groups after 4 days as the post-germination stage. The total RNA (5 µg) of each replicate was extracted from the bulk seed sample, including all seed parts, using Trizol (Thermo Fisher, cat. 15596018). High-quality RNA samples with an RNA integrity number (> 7.0) by 2100 Bioanalyzer (Agilent, cat. G2939A) and RNA 6000 Nano Kit (Agilent, cat. 5067-1511) were used to construct the sequencing library. One replicate was excluded from the control group due to quality control failure. The mRNA of each sample was purified from total RNA using Dynabeads™ Oligo(dT) (Thermo Fisher, cat. 61021). The purified mRNA was reduced into short fragments by the Magnesium RNA Fragmentation Module (NEB, cat. E6150). The cleaved RNA fragments were reverse-transcribed to create cDNA by SuperScript II Reverse Transcriptase (Invitrogen, cat. 1896649), which was used to synthesize U-labeled second-stranded DNAs with *E. coli* DNA polymerase I (NEB, cat. M0209), RNase H (NEB, cat. M0297), and dUTP solution (Thermo Fisher, cat. R0133). An A-base was added to the 3′ ends of each strand for ligating to the indexed adapters. Each adapter contained a T-base overhang for ligating to the A-tailed fragmented DNA. Dual-index adapters were ligated to the fragments, and size selection was performed with the AMPure XP beads. After the heat-labile UDG enzyme (NEB, cat. M0280) treatment of the U-labeled second-stranded DNAs, the ligated products were amplified with PCR. The average insert size for the final cDNA library was 300 ± 50 bp. The cDNA library was sequenced with the Illumina NovaSeq™ 6000 platform, generating a total of million 2 × 150 bp paired-end (PE150) reads. Cutadapt (V. 1.9)^68^ removed reads with adapters, poly(A) and poly(G), unknown nucleotides greater than 5%, and low-quality bases greater than 20%. The sequence quality was verified with FastQC (V. 0.11.9)^69^, and clean reads were generated as FASTQ files.

The reads of all samples were mapped to the lettuce reference genome (V. 8.0) using HISAT2 (V. 2.0.4)^70^. The mapped reads of each sample were assembled using StringTie (V. 1.3.4d)^71^. Transcripts from all samples were merged to reconstruct a comprehensive transcriptome using GffCompare (V. 0.9.8)^72^. After the final transcriptome was generated, StringTie and Ballgown^73^ were used to estimate the expression levels of all transcripts and perform expression abundance for mRNAs by calculating FPKM. The DEG analysis between the control and treatment datasets was performed using DESeq2 with the FPKM data^74^. The genes satisfying two criteria, *Q* < 0.05 (H_0_: log_2_(fold change) = 0) and absolute log_2_(fold change) ≥ 1, were considered DEGs.

### Enrichment analysis

The genes with GO terms and KEGG pathways were annotated in the GO (http://www.geneontology.org/)^75^ and KEGG (https://www.kegg.jp/kegg/)^76^ database. The KEGG identifiers combined with ko and 5-digit numbers were collected for the KEGG pathway. The enrichment analysis was conducted to identify the top 20 GO terms and KEGG pathways by the hypergeometric test. The *P*-value formula of the hypergeometric test was as follows: *P*-value = 1 – $$\sum\nolimits_{{i = 0}}^{{m - 1}} {{{\left( {\begin{array}{*{20}c} m \\ i \\ \end{array} } \right)\left( {\begin{array}{*{20}c} {N - M} \\ {n - i} \\ \end{array} } \right)} \mathord{\left/ {\vphantom {{\left( {\begin{array}{*{20}c} m \\ i \\ \end{array} } \right)\left( {\begin{array}{*{20}c} {N - M} \\ {n - i} \\ \end{array} } \right)} {\left( {\begin{array}{*{20}c} M \\ i \\ \end{array} } \right)}}} \right. \kern-\nulldelimiterspace} {\left( {\begin{array}{*{20}c} M \\ i \\ \end{array} } \right)}}}$$ (*N*: total background gene numbers annotated with all GO terms or KEGG pathways, *M*: background gene numbers annotated with a GO term or KEGG pathway, *n*: total significant DEG numbers, and *m*: significant DEG numbers in a GO term or KEGG pathway). The rich factor was calculated by dividing *m* by *M* from the *P*-value formula.

### Network analysis

The R source codes and terminologies of the weighted gene co-expression network analysis (WGCNA)^77^ were applied to elucidate the gene networks of seed germination in US96UC23. The FPKM data was transformed by log_2_(FPKM + 1). Genes with either 0 or excessive missing FPKM values were discarded based on the zero-variance criterion. The consensus network analysis was performed using the control (dH_2_O) and treatment (10% PEG) datasets. The weighted network was constructed as an unsigned network by raising the absolute correlation coefficient between a pair of genes to *β*. The modules were identified by hierarchical clustering, using the topological overlap matrix (TOM)-based dissimilarity and Dynamic Tree Cut. Each ME was extracted as the first principal component in a module. In the WGCNA^77^, eigengene significance, module significance, MM, and GS were defined as follows: (1) eigengene significance was the correlation coefficient between the germination percentage and ME in a module, (2) module significance was the average absolute GS for all genes in a module, (3) MM was the correlation coefficient between a gene and ME in a module, and (4) GS was the correlation coefficient between germination percentage and a gene. The liberal criterion (*P* < 0.05) was applied to test eigengene significance, MM, and GS. Eigengene significance and MM were tested in each dataset. GS was tested by the *P*-value in each dataset and the meta *P*-value across both datasets. Eigengene significance was evaluated to identify significant modules for constructing gene networks. Cytoscape^78^ was used to visualize the edge (interaction between a pair of genes) and node (gene) list files from WGCNA, to sort the top 20 hub genes by node degree in a module, and to estimate gene network parameters, betweenness centrality and neighborhood connectivity.

### Candidate genes

The coding sequences of lettuce genes were available in the Lettuce Genome Resource (V. 8.0) (https://lgr.genomecenter.ucdavis.edu/Home.php). The candidate genes for QTL were screened in the 95% CIs (± 1 LOD interval) of QTL. TAIR BLASTN (V. 2.9.0+) (https://www.arabidopsis.org/Blast/) was used to identify *A. thaliana* genes homologous to candidate genes in lettuce. The Bit score (> 50) and E-value (< 0.05) were used as criteria to find out the best identities.

## Electronic Supplementary Material

Below is the link to the electronic supplementary material.

Supplementary Material 1

Supplementary Material 2

Supplementary Material 3

Supplementary Material 4

Supplementary Material 5

Supplementary Material 6

Supplementary Material 7

Supplementary Material 8

## Data Availability

This published article and supplementary information files include all data generated or analyzed during this study. The raw RNA sequence data (FASTQ files) for transcriptome analysis is available in the European Nucleotide Archive (ENA) with accession number PRJEB71667. The corresponding author can provide additional data upon reasonable request.
