# Supplementary material for: QTL mapping and transcriptome analysis of seed germination under PEG-induced water stress in Lactuca spp
Source: Sci Rep. 2024 Nov 7;14:27157. doi: 10.1038/s41598-024-77972-9 (PMC11543936; doi:10.1038/s41598-024-77972-9)
Supplement: Supplementary file 8 — Supplementary Material 8 [file 41598_2024_77972_MOESM8_ESM.pdf]

# QTL mapping and transcriptome analysis of seed germination under PEG-induced water stress in *Lactuca* spp.

**Sadal Hwang[1,*], Ivan Simko[1], and Beiquan Mou[1]**

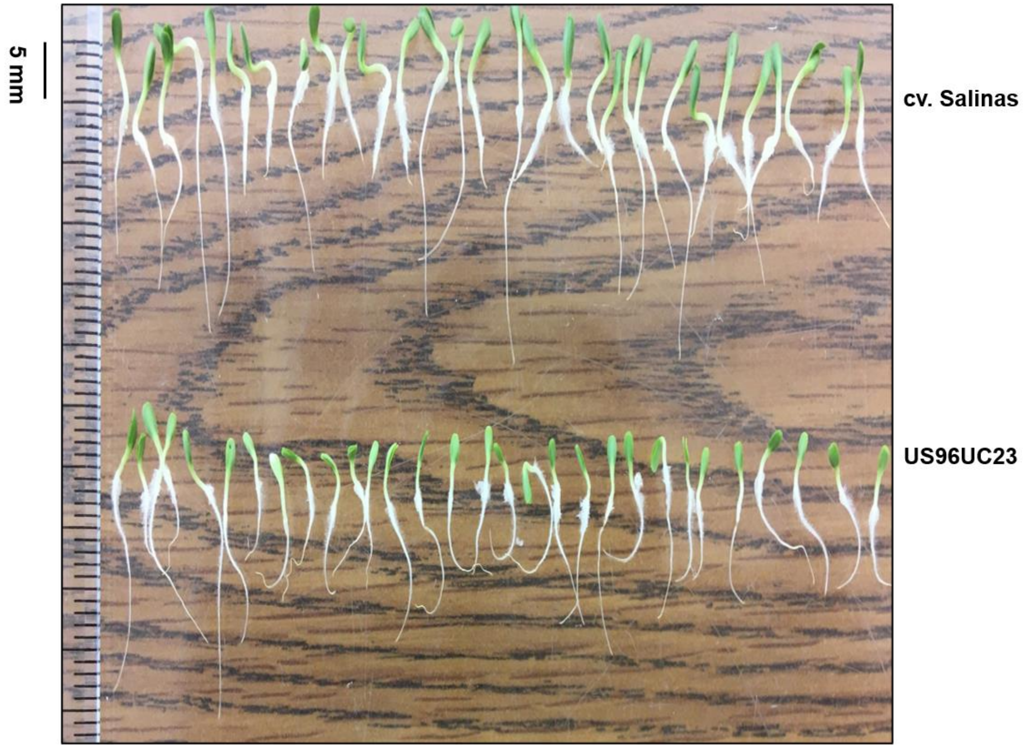


**Supplementary Fig. 1**. Seed germination of cv. Salinas and US96UC23 under control conditions. The photo showed the second replicate of 30 seeds from cv. Salinas and US96UC23 at $dH_2O$ after 4 days.

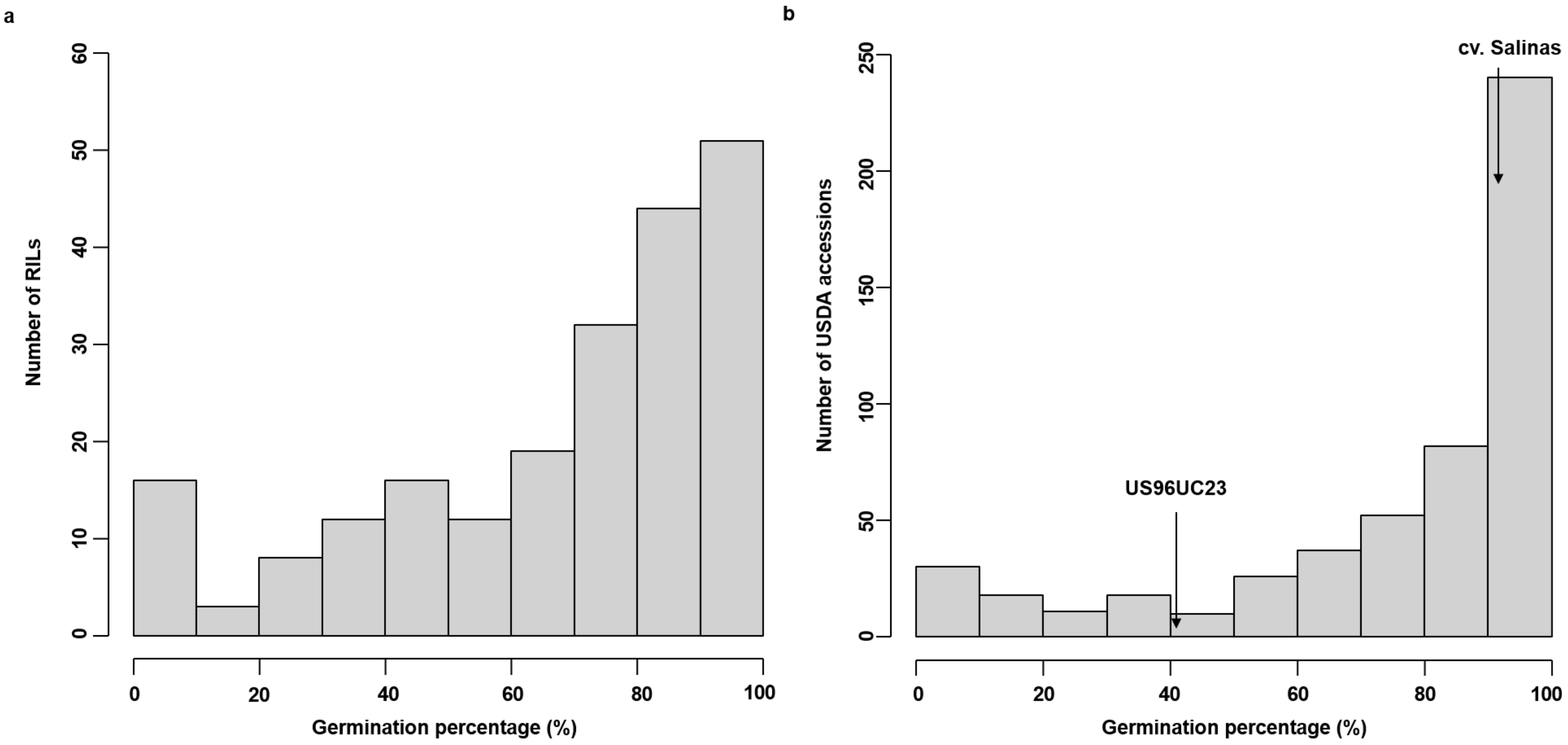


**Supplementary Fig. 2.** a. Histogram of germination percentage in the 213 cv. Salinas x US96UC23 RIL population at 10% PEG. b. Histogram of germination percentage in the 524 USDA germplasm collection at 10% PEG. Black arrows indicated the germination percentages of cv. Salinas and US96UC23, which were included in the USDA germplasm collection.

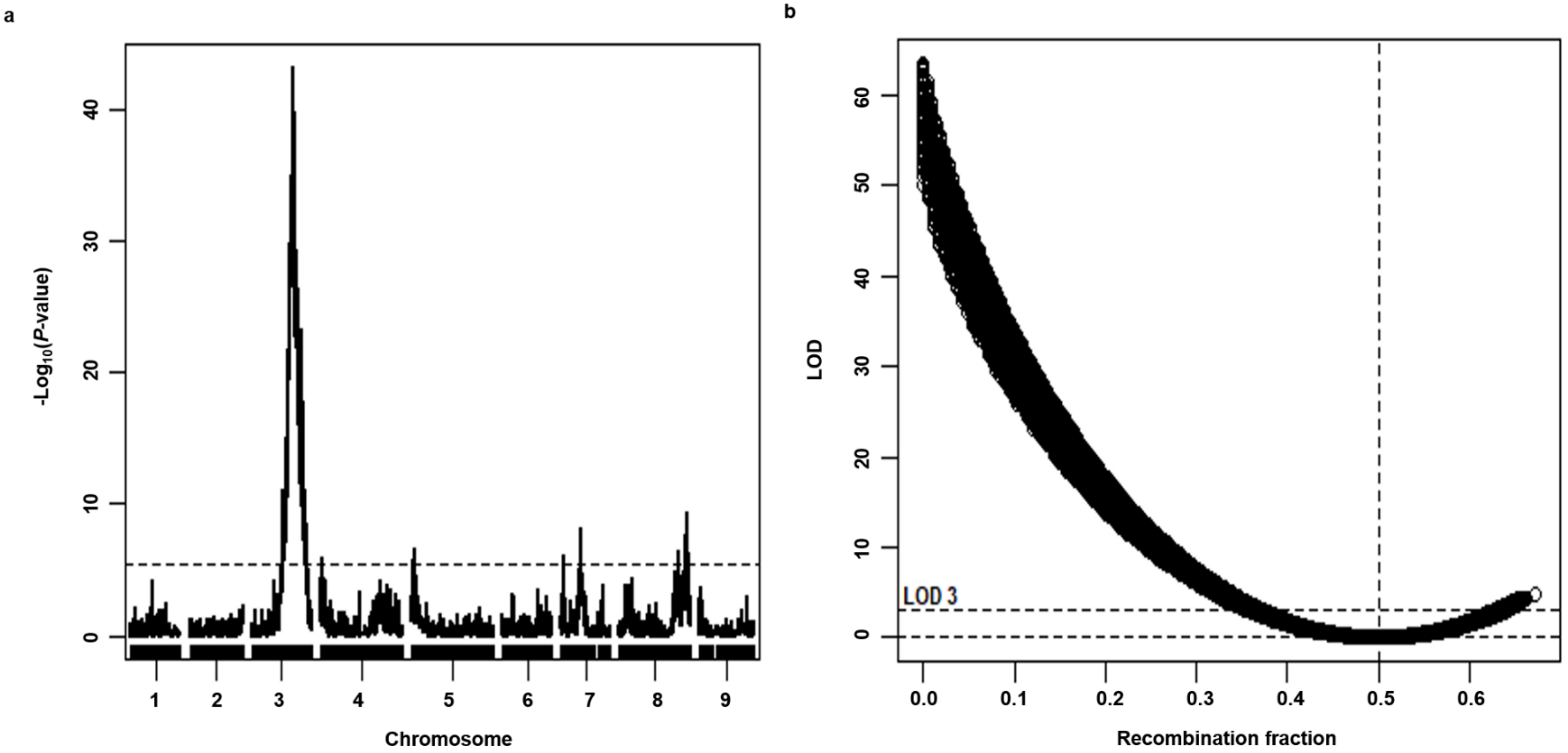


**Supplementary Fig. 3.** a. Identification of segregation-distorted markers. The y-axis indicated -$\log_{10}$(*P*-value), where the *P*-value came from the Chi-squared test of each marker. The horizontal dotted line intersected the y-axis at 5.45, equal to $-\log_{10}$(3.58e-06) as a Bonferroni-adjusted criterion. b. Relationship between *rf* and LOD in all SPP pairs. The LOD ($H_0$: *rf* = 0.5) of the horizontal dotted line was 3, and the *rf* of the vertical dotted line was 0.5. A downward spiking of *rf* reflected the linkage on a given chromosome, showing that the lower the *rf*, the higher the LOD. Some SPP pairs had *rf* greater than 0.5, and their pairs had LOD greater than 3, indicating that an upward spiking of *rf* was diagnostic of potential switched alleles.

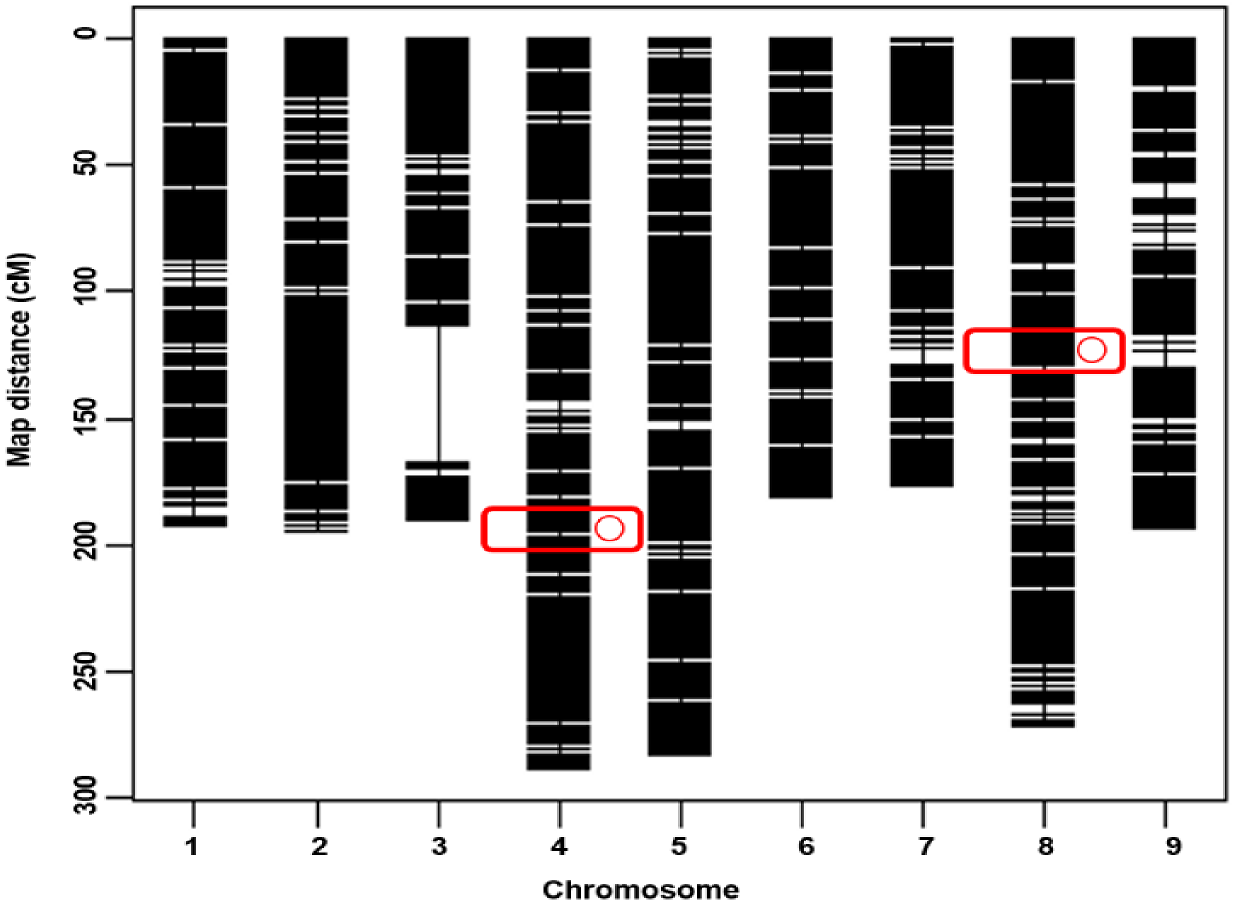


**Supplementary Fig. 4.** Final genetic map and QTL positions in the cv. Salinas × US96UC23 RIL population. The red dots in red rectangles indicated QTL positions.

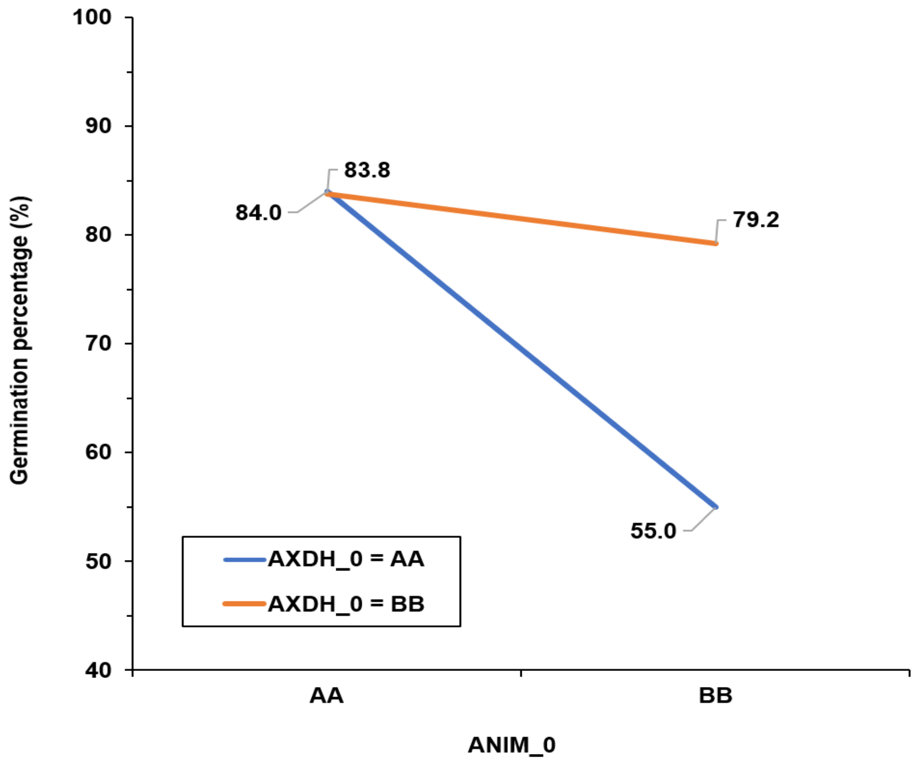


**Supplementary Fig. 5.** Germination percentages of four genotype groups, AA/AA, AA/BB, BB/AA, and BB/BB, respectively. The A and B alleles originated from cv. Salinas and US96UC23, respectively. Each genotype group comprised the first two alleles from ANIM_0 and the last two from AXDH_0. The line graph displayed the average germination percentages of the four genotype groups.

| Chromosome | Gene ID[a] | Gene name[b] | Position[c] | Gene name[d] | *A. thaliana* gene description |
|---|---|---|---|---|---|
| 4 | **LS10613** | **Lsat_1_v5_gn_4_126860.1** | 233280539-233281919 | AT4G36930.1 | Basic helix-loop-helix (bHLH) DNA-binding superfamily |
| | **LS10615** | **Lsat_1_v5_gn_4_126840.1** | 233707311-233710157 | AT4G02630.1 | Protein kinase superfamily |
| | **NA** | **Lsat_1_v5_gn_4_127141.1** | 234247544-234248403 | AT5G52170.3 | Homeodomain glabrous 7 |
| | **LS10616** | **Lsat_1_v5_gn_4_127120.1** | 234315308-234316721 | AT1G46480.2 | WUSCHEL-related homeobox 4 |
| | **NA** | **NA** | 234370421-234371251 | AT5G21280.1 | Hydroxyproline-rich glycoprotein family |
| | **LS10617** | **Lsat_1_v5_gn_4_127080.1** | 234423423-234424351 | AT1G46768.3 | ERF/AP2 transcription factor family |
| | **NA** | **Lsat_1_v5_gn_4_127040.1** | 234626399-234629375 | AT3G20290.3 | EPS15 homology domain |
| | **NA** | **Lsat_1_v5_gn_4_127480.1** | 234812307-234813054 | AT2G47070.2 | Squamosa promoter binding protein-like 1 |
| | **LS10618** | **Lsat_1_v5_gn_4_127421.1** | 234879311-234883202 | AT3G10360.1 | Pumilio (APUM) 4 |
| | **LS10619** | **Lsat_1_v5_gn_4_127260.1** | 235026757-235028401 | AT2G38240.1 | 2-oxoglutarate (2OG) and Fe (II)-dependent oxygenase superfamily |
| | **NA** | **Lsat_1_v5_gn_4_127280.1** | 235123901-235128250 | AT3G54830.2 | Transmembrane amino acid transporter family |
| | **NA** | **Lsat_1_v5_gn_4_127381.1** | 235545908-235546258 | AT1G67000.1 | Protein kinase superfamily |
| | **NA** | **Lsat_1_v5_gn_4_127361.1** | 235548035-235548295 | AT2G28490.1 | RmlC-like cupins superfamily |
| | **LS10620** | **Lsat_1_v5_gn_4_127200.1** | 235726379-235727964 | AT5G52010.1 | $C_2H_2$-like zinc finger |
| | **NA** | **Lsat_1_v5_gn_4_127021.1** | 236087073-236090403 | AT5G42210.1 | Major facilitator superfamily |
| | **LS10621** | **Lsat_1_v5_gn_4_127620.1** | 236684350-236693360 | AT5G47820.2 | P-loop containing nucleoside triphosphate hydrolases superfamily |
| | **LS10622** | **Lsat_1_v5_gn_4_127841.1** | 236888355-236890152 | AT5G43020.1 | Leucine-rich repeat protein kinase family |
| | **NA** | **Lsat_1_v5_gn_4_127900.1** | 237081023-237081413 | AT5G22460.3 | Alpha/beta-hydrolases superfamily |
| | **LS10623** | **Lsat_1_v5_gn_4_127960.1** | 237326727-237328801 | AT2G23320.1 | WRKY DNA-binding protein |
| | **LS10624** | **Lsat_1_v5_gn_4_127800.1** | 237723236-237724146 | AT3G16610.1 | Pentatricopeptide (PPR) repeat-containing protein |
| | **LS10625** | **Lsat_1_v5_gn_4_127821.1** | 237724979-237725636 | AT1G63310.1 | NA - hypothetical protein |
| | **LS10626** | **Lsat_1_v5_gn_4_127640.1** | 237902680-237905864 | AT5G43060.1 | Granulin repeat cysteine protease family |
| | **LS10627** | **Lsat_1_v5_gn_4_127661.1** | 237906393-237907382 | AT2G23310.2 | Rer1 family |
| | **NA** | **Lsat_1_v5_gn_4_127701.1** | 238012481-238012738 | AT5G64080.2 | Bifunctional inhibitor/lipid-transfer protein / seed storage 2S albumin superfamily |
| | **LS10628** | **Lsat_1_v5_gn_4_127741.1** | 238017737-238019631 | AT5G67250.1 | SKP1/ASK1-interacting protein 2 |
| 8 | **LS21896** | **Lsat_1_v5_gn_8_85621.1** | 124464107-124469258 | AT4G35740.1 | DEAD/DEAH box RNA helicase family |
| | **LS21897** | **Lsat_1_v5_gn_8_85600.1** | 124467301-124469258 | AT3G59530.3 | Calcium-dependent phosphotriesterase superfamily |
| | **LS21898** | **Lsat_1_v5_gn_8_85581.1** | 124472616-124475031 | AT3G14070.1 | Cation exchanger 9 |
| | **LS21899** | **Lsat_1_v5_gn_8_85560.1** | 124480573-124482649 | AT2G05940.1 | Protein kinase superfamily |
| | **NA** | **Lsat_1_v5_gn_8_85541.1** | 124494663-124495040 | AT2G22360.1 | DNAJ heat shock family protein |
| | **NA** | **Lsat_1_v5_gn_8_86500.1** | 124558287-124558625 | AT2G20370.1 | Exostosin family protein |

| | | | | |
|---|---|---|---|---|
| **LS21900** | **Lsat_1_v5_gn_8_86481.1** | 124598388-124600996 | AT2G06005.1 | FRIGIDA interacting protein 1 (FIP 1) |
| **LS21901** | **Lsat_1_v5_gn_8_86460.1** | 124600590-124605092 | AT1G77550.1 | Tubulin-tyrosine ligase |
| **LS21902** | **Lsat_1_v5_gn_8_86441.1** | 124642734-124644123 | AT5G56550.1 | Oxidative stress 3 (OXS 3) |
| **LS21903** | **Lsat_1_v5_gn_8_86420.1** | 124645554-124648271 | AT5G45550.1 | MOB1/phocein family |

**Supplementary Table 1.** Candidate genes associated with QTL for germination percentage at 10% PEG in the cv. Salinas × US96UC23 RIL population. [a]The lettuce gene IDs consisted of the prefix LS followed by a 5-digit number from a pool of 27,245 genes (Supplementary Data 2). They were used to describe lettuce genes instead of long gene model names in the lettuce reference genome (V. 8.0). The NA stands for not applicable. [b]Lettuce gene model name annotated in the lettuce reference genome (V. 8.0) from the Lettuce Genome Resource (https://lgr.genomecenter.ucdavis.edu/Home.php). [c]Physical map position of lettuce gene in the lettuce reference genome (V. 8.0). [d]*A. thaliana* gene model name designated by TAIR (https://www.arabidopsis.org/).

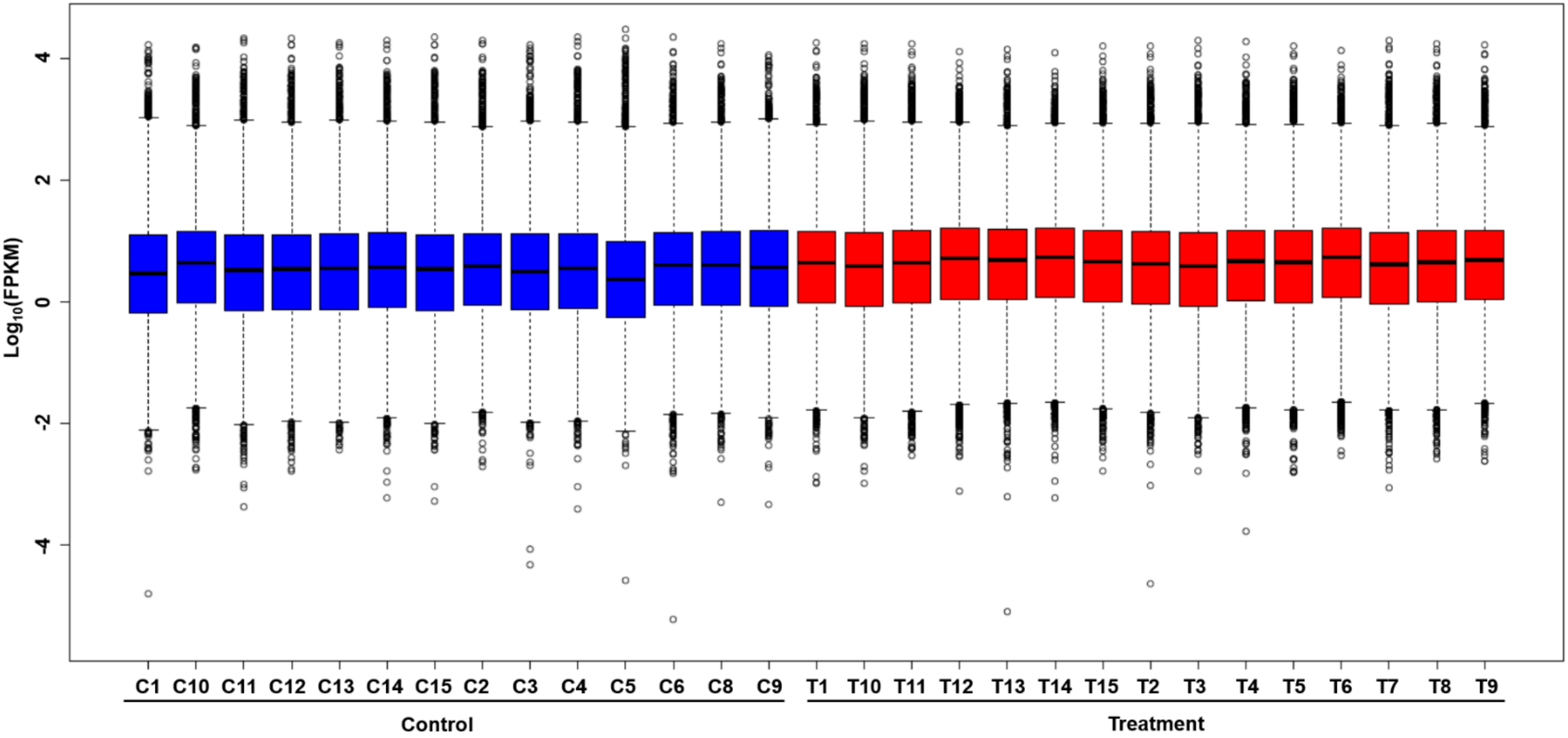


**Supplementary Fig. 6.** Distribution of $log_{10}$(FPKM) in the control ($dH_2O$) and treatment (10% PEG) datasets. The distributions of all replicates excluded the data points with FPKM equal to zero.

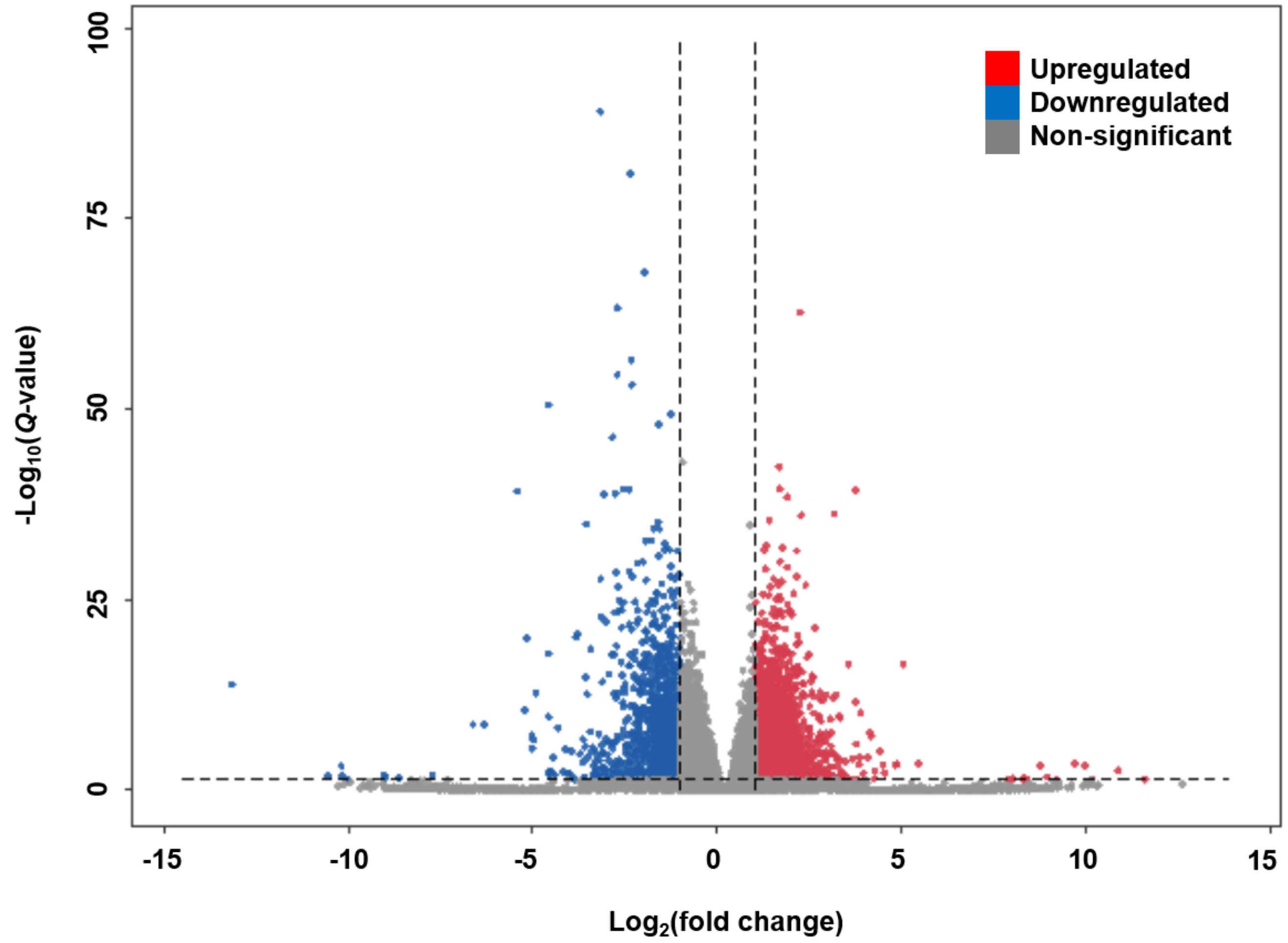


**Supplementary Fig. 7.** Screening of significant DEGs. The red, blue, and grey data points indicated upregulated, downregulated, and non-significant DEGs. The vertical dotted lines intersected the x-axis at -1 and 1. The $-\log_{10}$($Q$-value) value of the horizontal dotted line was 1.3.

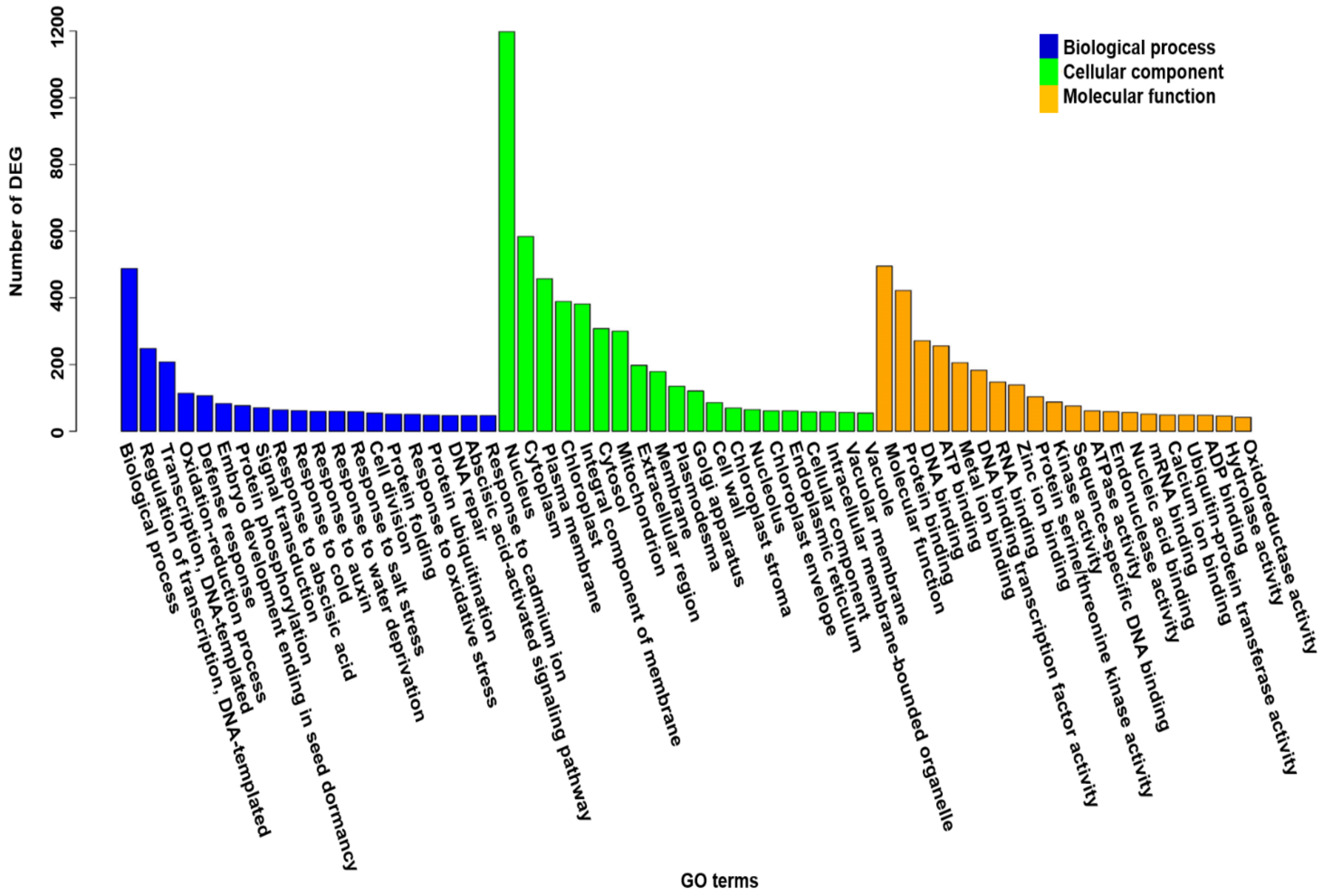


**Supplementary Fig. 8.** Top 20 GO terms annotated in the largest number of DEGs in three GO classes.

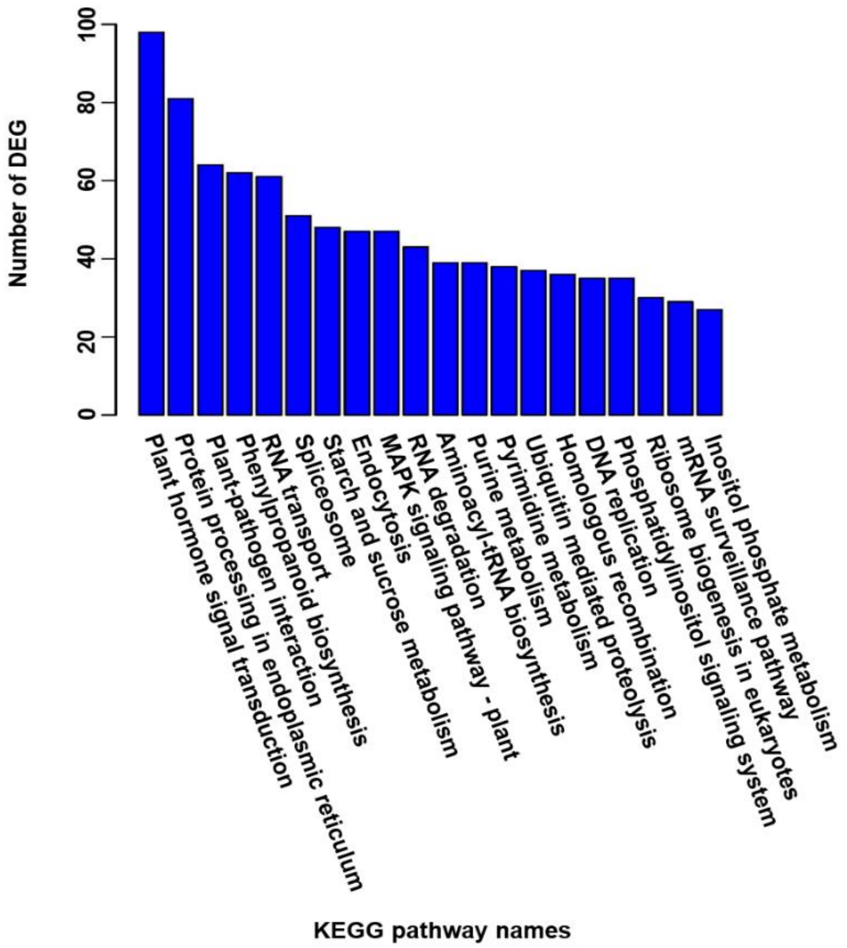


**Supplementary Fig. 9.** Top 20 KEGG pathways annotated in the largest number of DEGs.

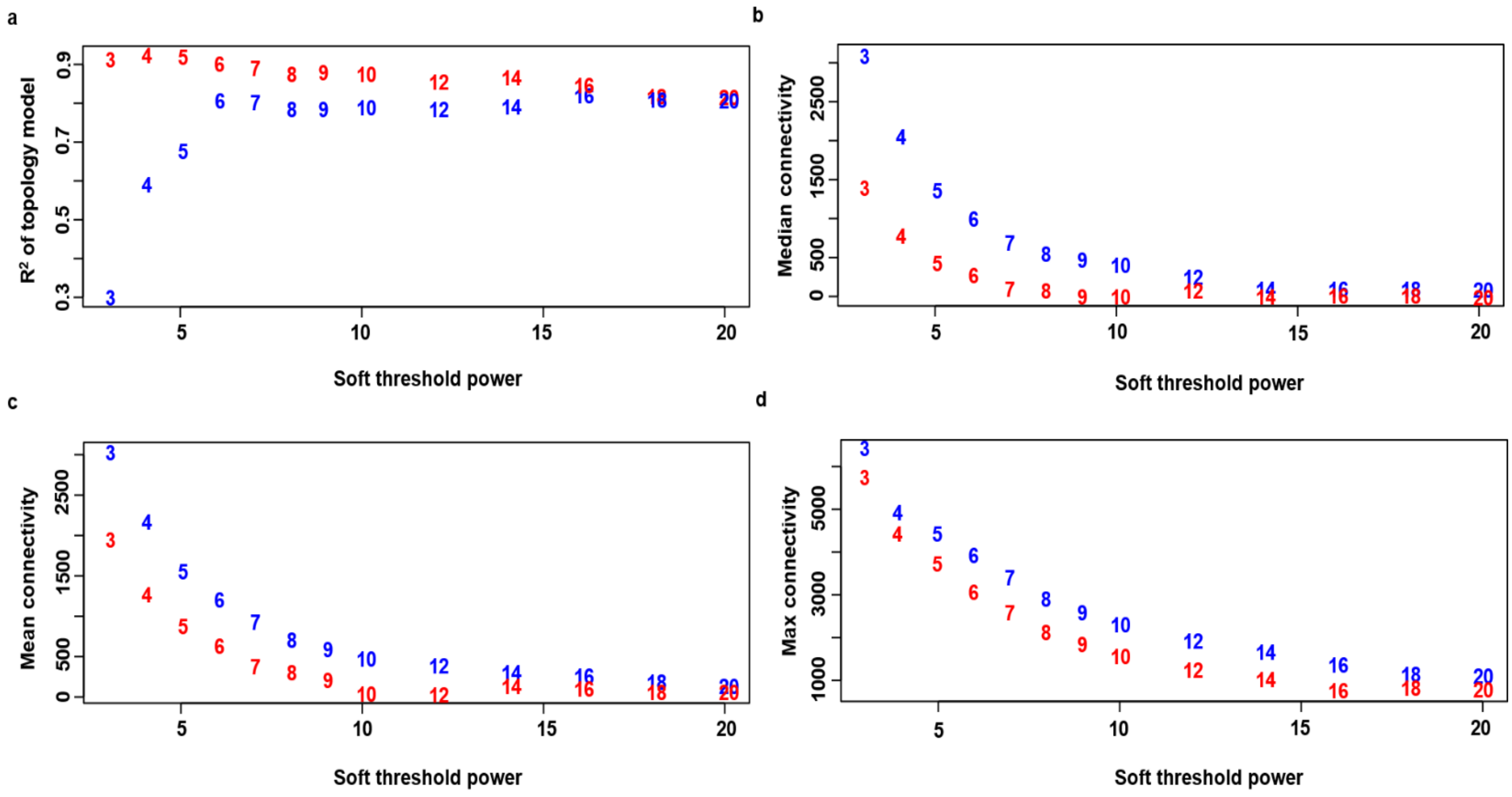


**Supplementary Fig. 10.** Determination of soft threshold power, $\beta$. The numbers in red and blue indicated $\beta$ in the control and treatment datasets, respectively. a. $R^2$ values of topology model. b. Median connectivity. c. Mean connectivity. d. Max connectivity.

| Module[a] | Eigengene significance | | | | Module significance | | Module membership[b] | |
|---|---|---|---|---|---|---|---|---|
| | Control | | Treatment | | Control | Treatment | Control | Treatment |
| | | *P*-value | | *P*-value | | | | |
| 0 | -0.6310 | 0.0137 | -0.0497 | 0.8631 | 0.4908 | 0.2021 | NA | NA |
| 1 | -0.1903 | 0.5229 | -0.5987 | 0.0167 | 0.1793 | 0.4162 | 0.6908 | 0.6567 |
| 2 | 0.0051 | 0.9864 | -0.5007 | 0.0566 | 0.1420 | 0.3140 | 0.6367 | 0.5504 |
| 3 | 0.0986 | 0.7428 | 0.2859 | 0.3082 | 0.1310 | 0.2432 | 0.7069 | 0.5935 |
| 4 | -0.1331 | 0.6571 | -0.6444 | 0.0080 | 0.1581 | 0.3902 | 0.7281 | 0.5829 |
| 5 | 0.0746 | 0.8043 | -0.1156 | 0.6875 | 0.1193 | 0.1956 | 0.7464 | 0.6428 |
| 6 | -0.0825 | 0.7839 | -0.6424 | 0.0083 | 0.1355 | 0.4005 | 0.6930 | 0.6186 |
| 7 | -0.1859 | 0.5328 | -0.4951 | 0.0601 | 0.1898 | 0.3347 | 0.6470 | 0.6094 |
| 8 | 0.0698 | 0.8166 | 0.0817 | 0.7766 | 0.1524 | 0.2191 | 0.6698 | 0.5775 |
| 9 | 0.1073 | 0.7210 | 0.5899 | 0.0189 | 0.1397 | 0.3860 | 0.7634 | 0.6474 |
| 10 | 0.1103 | 0.7133 | 0.3421 | 0.2170 | 0.1325 | 0.2806 | 0.7600 | 0.6853 |
| 11 | -0.0133 | 0.9648 | -0.3808 | 0.1648 | 0.1027 | 0.3010 | 0.7283 | 0.6731 |
| 12 | -0.2736 | 0.3518 | -0.4930 | 0.0614 | 0.2394 | 0.2306 | 0.7526 | 0.3185 |
| 13 | 0.0302 | 0.9202 | -0.2806 | 0.3179 | 0.1754 | 0.2312 | 0.6376 | 0.7460 |
| 14 | -0.0497 | 0.8689 | -0.0451 | 0.8758 | 0.1133 | 0.1549 | 0.7687 | 0.7075 |
| 15 | 0.1351 | 0.6521 | -0.0565 | 0.8448 | 0.1634 | 0.1442 | 0.7141 | 0.7001 |
| 16 | 0.0704 | 0.8150 | 0.2335 | 0.4099 | 0.1143 | 0.1983 | 0.7335 | 0.6879 |
| 17 | -0.2033 | 0.4940 | -0.5295 | 0.0412 | 0.2055 | 0.3285 | 0.7832 | 0.5776 |
| 18 | -0.0034 | 0.9910 | -0.0099 | 0.9728 | 0.1634 | 0.1257 | 0.8027 | 0.7838 |
| 19 | 0.0450 | 0.8813 | -0.5769 | 0.0227 | 0.1163 | 0.3963 | 0.7462 | 0.6861 |
| 20 | 0.0768 | 0.7985 | 0.1807 | 0.5269 | 0.1095 | 0.2114 | 0.7503 | 0.6978 |
| 21 | 0.0513 | 0.8649 | 0.0251 | 0.9307 | 0.1516 | 0.1388 | 0.7275 | 0.7040 |
| 22 | 0.1113 | 0.7109 | 0.4103 | 0.1309 | 0.1407 | 0.2973 | 0.6500 | 0.7144 |
| 23 | 0.1674 | 0.5751 | -0.1726 | 0.5460 | 0.1985 | 0.1913 | 0.6055 | 0.7186 |
| 24 | 0.1071 | 0.7215 | -0.5378 | 0.0373 | 0.1551 | 0.3983 | 0.7237 | 0.7467 |
| 25 | 0.1446 | 0.6292 | -0.2541 | 0.3681 | 0.1826 | 0.2244 | 0.5881 | 0.7202 |
| 26 | -0.0020 | 0.9947 | -0.1503 | 0.5999 | 0.1586 | 0.1701 | 0.6892 | 0.6614 |
| 27 | 0.0426 | 0.8875 | 0.2110 | 0.4581 | 0.1091 | 0.2062 | 0.7697 | 0.7124 |
| 28 | 0.0773 | 0.7974 | 0.2549 | 0.3666 | 0.1762 | 0.2101 | 0.6387 | 0.6839 |
| 29 | 0.0773 | 0.7972 | -0.2337 | 0.4096 | 0.1554 | 0.2008 | 0.6223 | 0.7111 |
| 30 | 0.0382 | 0.8991 | 0.0655 | 0.8203 | 0.1109 | 0.1539 | 0.7533 | 0.7243 |
| 31 | 0.0871 | 0.7721 | -0.2527 | 0.3709 | 0.1194 | 0.2009 | 0.7343 | 0.6930 |
| 32 | 0.1242 | 0.6788 | 0.1073 | 0.7090 | 0.1427 | 0.2075 | 0.8080 | 0.7491 |

| | | | | | | | | |
|---|---|---|---|---|---|---|---|---|
| 33 | 0.0142 | 0.9624 | 0.1069 | 0.7100 | 0.1604 | 0.1511 | 0.6772 | 0.6353 |
| 34 | -0.0069 | 0.9817 | -0.1575 | 0.5823 | 0.1095 | 0.1986 | 0.7960 | 0.6824 |
| 35 | 0.1269 | 0.6721 | 0.4181 | 0.1229 | 0.1552 | 0.3189 | 0.6765 | 0.7545 |
| 36 | -0.1030 | 0.7318 | 0.6888 | 0.0034 | 0.1190 | 0.5179 | 0.7964 | 0.7558 |
| 37 | -0.0857 | 0.7758 | -0.6333 | 0.0097 | 0.1461 | 0.3703 | 0.7745 | 0.5661 |
| 38 | 0.1074 | 0.7207 | 0.7908 | 0.0002 | 0.1394 | 0.5269 | 0.7092 | 0.6800 |
| 39 | -0.0824 | 0.7841 | -0.4761 | 0.0728 | 0.1433 | 0.3270 | 0.7840 | 0.6397 |
| 40 | -0.0045 | 0.9881 | -0.4568 | 0.0875 | 0.0997 | 0.3403 | 0.7572 | 0.7476 |
| 41 | 0.0457 | 0.8796 | -0.2898 | 0.3014 | 0.1718 | 0.2447 | 0.6098 | 0.7142 |
| 42 | -0.1144 | 0.7032 | -0.1566 | 0.5845 | 0.1252 | 0.1782 | 0.7969 | 0.7491 |
| 43 | 0.0575 | 0.8486 | -0.4774 | 0.0719 | 0.1569 | 0.3461 | 0.7054 | 0.6963 |
| 44 | -0.1514 | 0.6127 | -0.1679 | 0.5570 | 0.1471 | 0.2080 | 0.7186 | 0.6229 |

**Supplementary Table 2.** Estimation of eigengene significance, module significance, and module membership in the control and treatment datasets. [a]Module 0 was nominally assigned to 48 genes in the grey-colored module. Therefore, the MM of module 0 was not applicable (NA). [b]The average absolute MM of all genes in each module was estimated. The sign of MM in each gene was not presented in the table.

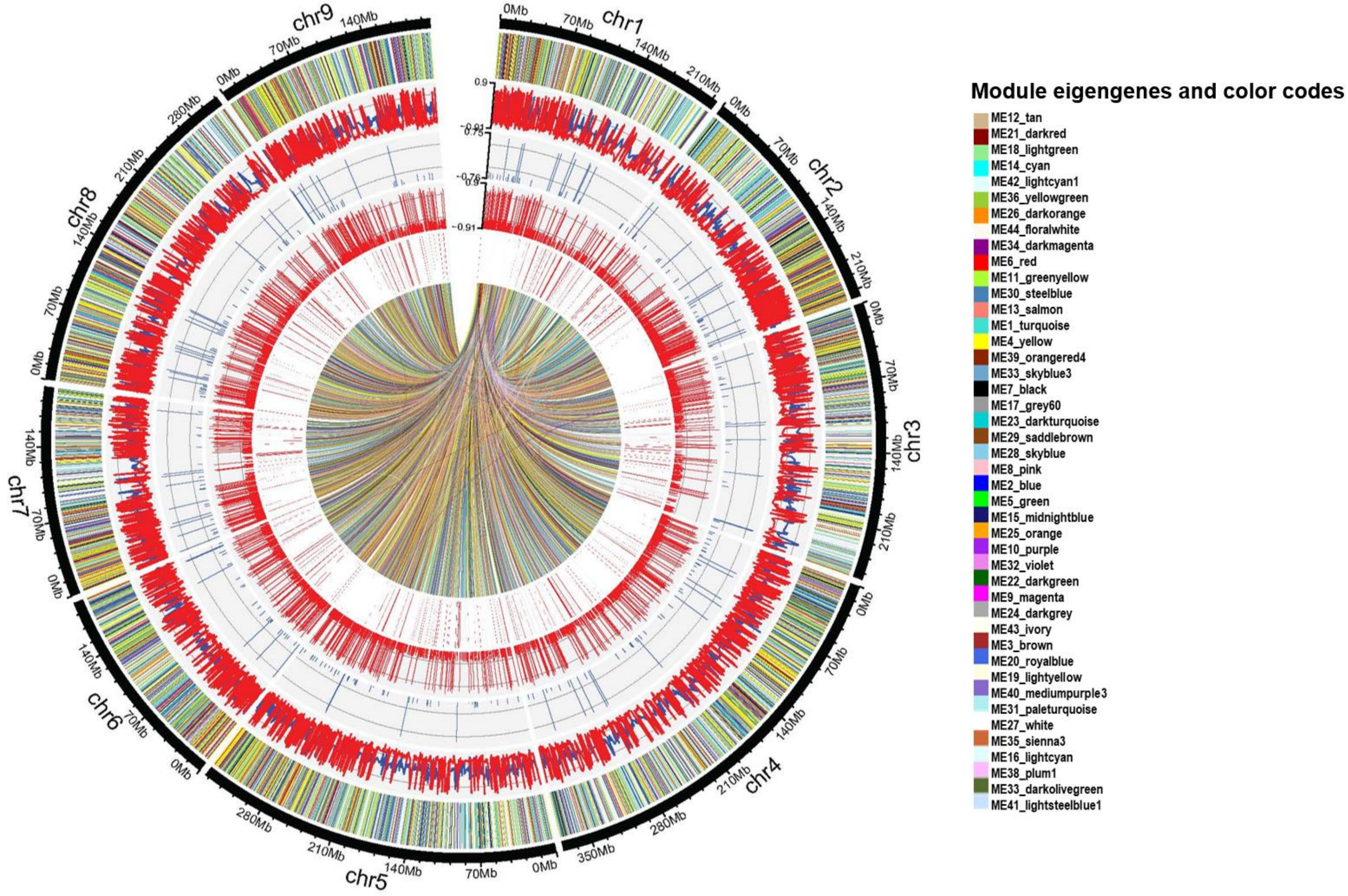


**Supplementary Fig. 11.** Identification of significant 6,378 genes in GS. Each track information in Circos plot was as follows: 1) first track: general chromosome information, 2) second track: assigned module colors for 6,378 genes on each chromosome, 3) third track: blue and red lines indicated significant genes by *P*-value or meta *P*-value in the control and treatment datasets, 4) fourth track: blue bars indicated significant genes by *P*-value in the control dataset, 5) fifth track: red bars indicated significant genes by *P*-value in the treatment dataset, 6) sixth track: red lines indicated DEGs, and 7) Link: links of the same modules among chromosomes.

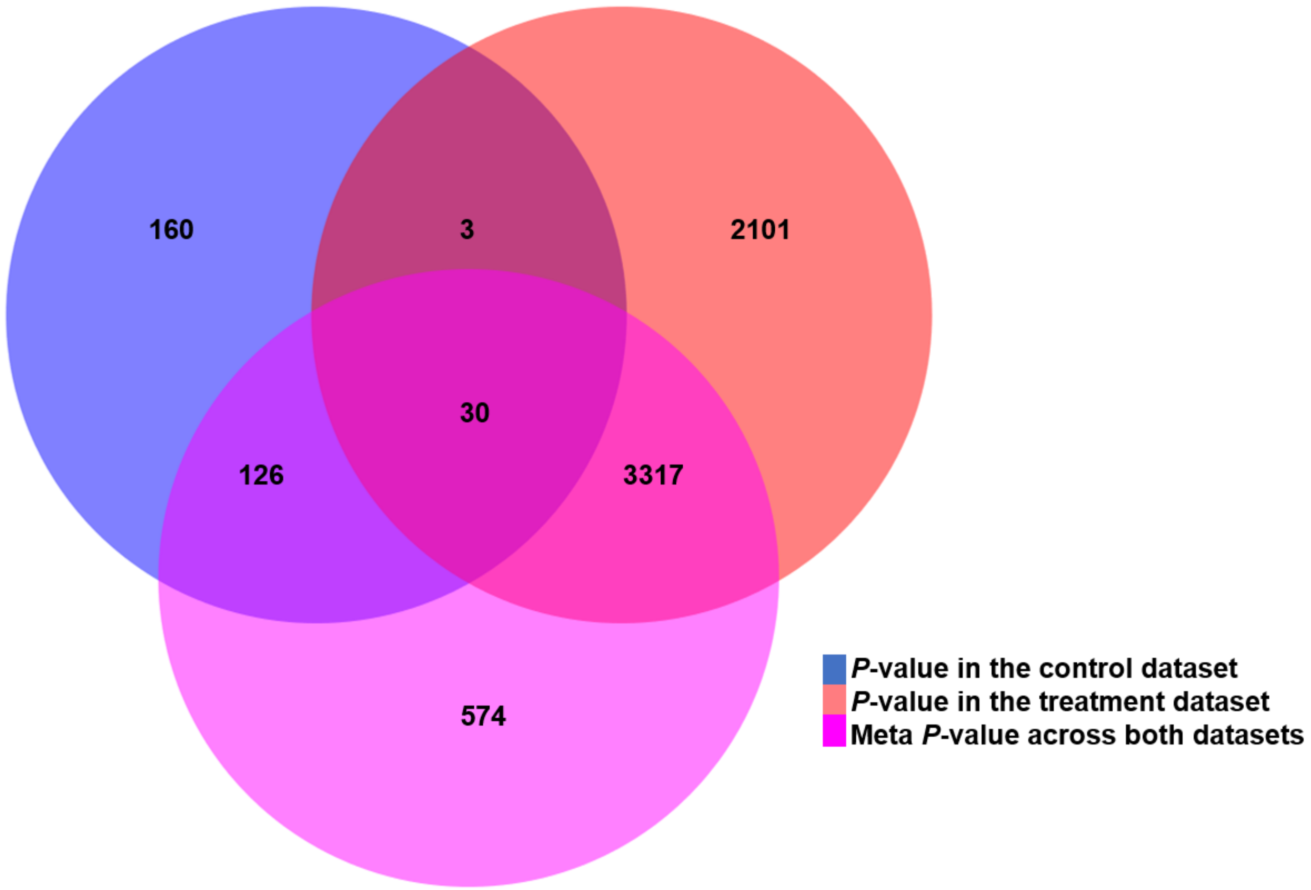


**Supplementary Fig. 12.** Venn diagram of significant genes according to the GS test.

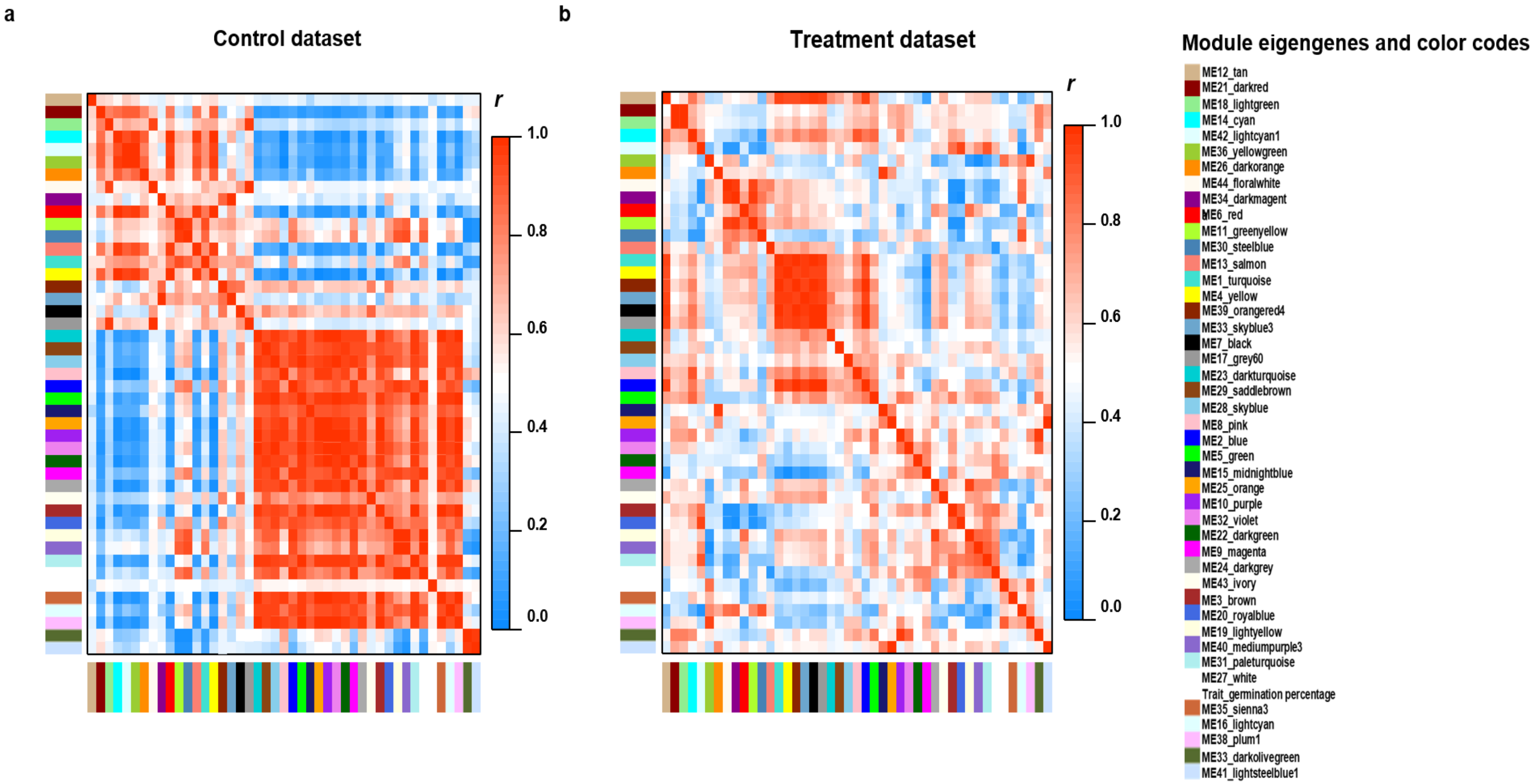


**Supplementary Fig. 13.** Relationships among MEs in the control and treatment datasets. The 44 MEs were used in each dataset. a. Correlation coefficients among MEs in the control dataset. b. Correlation coefficients among MEs in the treatment dataset. Both heatmaps used module colors instead of ME labels on the x- and y-axes.
